# Indoleamine-2,3-dioxygenase inhibition improves immunity and is safe for concurrent use with cART during Mtb/SIV coinfection

**DOI:** 10.1172/jci.insight.179317

**Published:** 2024-07-02

**Authors:** Bindu Singh, Riti Sharan, Gayathri Ravichandran, Ruby Escobedo, Vinay Shivanna, Edward J. Dick, Shannan Hall-Ursone, Garima Arora, Xavier Alvarez, Dhiraj K. Singh, Deepak Kaushal, Smriti Mehra

**Affiliations:** Texas Biomedical Research Institute, San Antonio, Texas, USA.

**Keywords:** Immunology, Infectious disease, Tuberculosis

## Abstract

Chronic immune activation promotes tuberculosis (TB) reactivation in the macaque *Mycobacterium tuberculosis* (*M. tuberculosis*)/SIV coinfection model. Initiating combinatorial antiretroviral therapy (cART) early lowers the risk of TB reactivation, but immune activation persists. Studies of host-directed therapeutics (HDTs) that mitigate immune activation are, therefore, required. Indoleamine 2,3, dioxygenase (IDO), a potent immunosuppressor, is one of the most abundantly induced proteins in NHP and human TB granulomas. Inhibition of IDO improves immune responses in the lung, leading to better control of TB, including adjunctive to TB chemotherapy. The IDO inhibitor D-1 methyl tryptophan (D1MT) is, therefore, a bona fide TB HDT candidate. Since HDTs against TB are likely to be deployed in an HIV coinfection setting, we studied the effect of IDO inhibition in *M. tuberculosis*/SIV coinfection, adjunctive to cART. D1MT is safe in this setting, does not interfere with viral suppression, and improves the quality of CD4^+^ and CD8^+^ T cell responses, including reconstitution, activation and *M. tuberculosis*–specific cytokine production, and access of CD8^+^ T cells to the lung granulomas; it reduces granuloma size and necrosis, type I IFN expression, and the recruitment of inflammatory IDO^+^ interstitial macrophages (IMs). Thus, trials evaluating the potential of IDO inhibition as HDT in the setting of cART in *M. tuberculosis*/HIV coinfected individuals are warranted.

## Abstract 2

Inhibition of IDO improves immune responses in the lung, leading to better control of tuberculosis, and does not interfere with viral suppression in the setting combinatorial antiretroviral therapy.

## Introduction

HIV coinfection predisposes the host to reactivation of latent tuberculosis infection (LTBI), resulting in tuberculosis (TB) reactivation. Studies using the nonhuman primate (NHP) model of *Mycobacterium tuberculosis* (*M. tuberculosis)*/SIV coinfection show that CD4^+^ T cell depletion due to SIV coinfections is insufficient in causing LTBI reactivation. Instead, SIV-induced chronic immune activation is critical for reactivation (1). While earlier introduction of combinatorial antiretroviral therapy (cART) significantly reduced the recruitment of myeloid cells expressing immunoregulatory proteins like indoleamine 2,3, dioxygenase (IDO) to the lung granulomas, facilitated T cell reconstitution, and better controlled TB reactivation (2), as opposed to late cART (3), chronic immune activation was not completely mitigated. Thus, it is particularly important to develop host-directed therapeutics (HDTs) that target chronic immune activation as interventions that can help mitigate TB reactivation in the setting of HIV coinfection.

Gene- (4–6) and protein-expression (7, 8) studies have revealed that human TB granulomas are enriched for IDO^+^ myeloid cells (7, 8). High levels of IDO products are also detected in patients with TB (8–11). IDO, which catabolizes the essential amino acid tryptophan (Trp) (12), is the most abundant protein in the myeloid layer of human and rhesus macaque (RM) TB lesions in an ordered spatial context (8, 13, 14). IDO affects T cell proliferation and promotes immunosuppression — e.g., by impairing TB control pathways like phagolysosome fusion and autophagy (15, 16) — inhibiting CD4^+^ T cell proliferation in the absence of Trp and expanding Tregs and MDSCs. This creates an immunosuppressive environment conducive to *M. tuberculosis* persistence (17, 18). IDO is induced in granulomas in mice, NHPs, and humans (8, 17). IDO blockade is therefore an attractive HDT target for TB, with potential to inhibit chronic immune activation while enhancing adaptive immune function. IDO expression in *M. tuberculosis*/SIV coinfection is highly correlated with chronic immune activation and TB reactivation (3). Treatment with D-1 methyl tryptophan (D1MT), a structural analog of Trp, inhibits IDO enzyme activity in *M. tuberculosis*–infected RMs (17), improving immune responses and reducing the burden of disease (17). IDO inhibition was also safe and effective in the presence of anti-TB chemotherapy (19).

Here, we concurrently treated *M. tuberculosis*/SIV–coinfected RMs with cART (for ~10 weeks) and D1MT (for 4 weeks), beginning at week 11 (2 weeks after SIV) and compared these with a historical control group of *M. tuberculosis*/SIV–coinfected RMs treated with cART at the same time (2) and with current as well as historical *M. tuberculosis*/SIV–coinfected controls (3, 20) (Supplemental Table 1; supplemental material available online with this article; https://doi.org/10.1172/jci.insight.179317DS1). We also included a control group where RMs were similarly monoinfected with SIVmac239 (Supplemental Table 1). We compared clinical attributes of progression to TB in these *M. tuberculosis*/SIV–coinfected RMs before, during, and after treatment; we compared PET/CT scans, morphometric lung pathology, bacterial burdens, immune cell dynamics including antigen-specific immune responses, and markers of immune activation. Our results show that inclusion of D1MT is safe in the setting of *M. tuberculosis*/SIV coinfection/cART and lowers chronic immune activation while enhancing adaptive anti-TB responses. Our results have the potential of furthering this HDT into clinical trials for people living with HIV (PLHIV) with LTBI.

## Results

### Survival and PET/CT-based disease evaluation of D1MT+cART-treated RMs relative to cART and M. tuberculosis/SIV–coinfected untreated controls.

**Figure 1:**
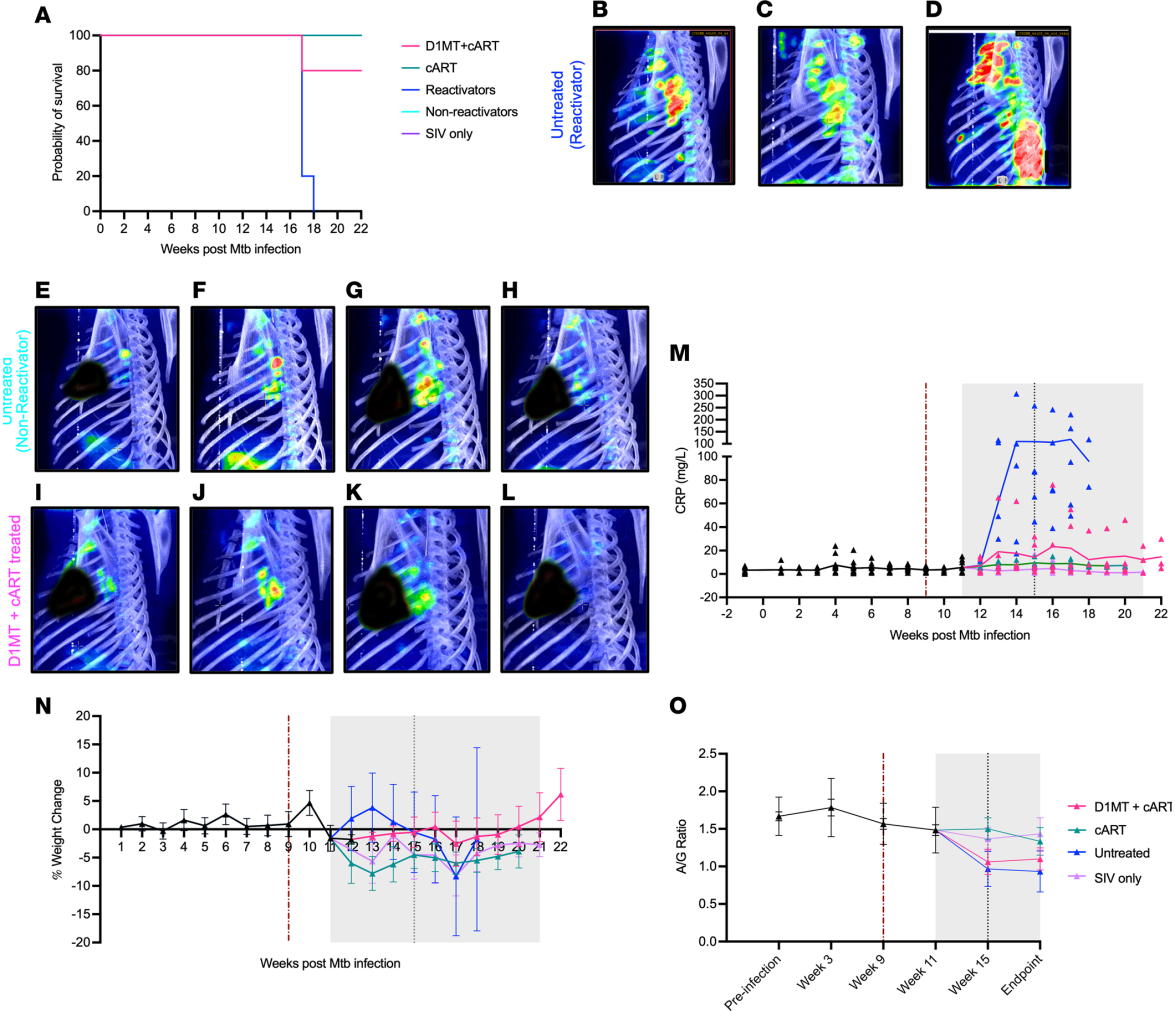
Survival characteristics and clinical correlates in D1MT+cART- relative to cART-treated *M. tuberculosis*/SIV–coinfected untreated control and SIV monoinfected RMs. (**A**) Survival curve depicting survival of RMs among the study groups. PET/CT scans were performed at wk6, wk10/11, wk16, and wk22/endpoint. (**B**–**L**) Shown are the representative PET/CT images from an untreated reactivator (**B**–**D**), an untreated nonreactivator (**E**–**H**), and a D1MT+cART-treated (**I**–**L**) RM at wk6 (**B**, **E**, and **I**), wk10/11 (**C**, **F**, and **J**), wk16 (**D**, **G**, and **K**), and wk22/endpoint (**H** and **L**). (**M**–**O**) Burgundy dotted line marks SIV infection in the coinfected groups, while the gray area represents cART phase and black dotted line represents D1MT treatment end. Data are represented as mean ± SEM. (**M**) Graphical representation of weekly serum CRP levels (mg/L) from the study start to the endpoint in D1MT+cART-treated, cART-treated, and untreated *M. tuberculosis*/SIV–coinfected as well as in SIV monoinfected animals. (**N**) Graph showing the percent weight change with respect to the baseline over the course of the study timeline. (**O**) Graphical representation of A/G ratios at different time points of the study.

We have earlier shown that coinfection with SIV in *M. tuberculosis*–infected RMs with latent TB infection (LTBI) results in reactivation of TB in the majority of animals within weeks (reactivators [Rs]), while some animals retain the immune control of *M. tuberculosis* in the time studied (nonreactivators [NRs]) (20–22). Early introduction of cART rapidly limits SIV replication and prevents clinical reactivation of TB, although SIV-induced chronic immune activation persists (2). Here we studied the effect of D1MT+cART treatment on the clinical progression of TB and SIV infection using various clinical attributes, including comparison of time-to-humane euthanasia. Only 1 of the 5 D1MT+cART-treated RMs progressed to euthanasia due to TB or SIV-related reasons during the protocol, and therefore, their survival was indistinguishable from either cART-treated RMs or NRs. None of the 4 RMs in the cART group required euthanasia prior to the completion of the protocol (Figure 1A). Of the *M. tuberculosis*/SIV–coinfected untreated animals, 5 RMs exhibited reactivation (consisting of historical controls and this study) and reached prespecified endpoints, resulting in the requirement of euthanasia prior to endpoint. Five untreated RMs did not exhibit clinical reactivation (consisting of historical controls and this study). Our results clearly show that inclusion of D1MT treatment does not alter the survival of *M. tuberculosis*/SIV–coinfected/cART-treated RMs. This is important since we have previously only reported treatment of RMs with D1MT, a generalized IDO inhibitor, which is known to induce Th1 responses, either as a monotherapy or adjunctive to anti-TB drugs (17, 19). We then used 18-FDG PET/CT, which we have previously described in detail (19, 23), as a means to quantify TB disease in *M. tuberculosis*/SIV–coinfected, cART-treated RMs with long-term D1MT administration. We compared 18-FDG PET/CT incorporation in the lungs of D1MT+cART-treated RMs with untreated R and NRs in the current study (Figure 1, B–L). PET/CT results were, however, not available for the cART-treated group or those historical untreated controls, which were involved in experiments in 2018–2020, before we acquired the PET/CT ability (2). Therefore, it was not possible to perform a direct comparison of the effect of D1MT concurrently with cART. Our results, however, clearly show progressively increasing TB disease levels in a representative, untreated *M. tuberculosis*/SIV–coinfected reactivator RM over time after SIV coinfection (Figure 1, B–D), as compared with a prototypical nonreactivator (Figure 1, E–H). All but 1 of the D1MT/cART-treated RMs exhibited PET/CT profiles comparable with nonreactivators (Figure 1, I–L). These results suggest that the extent of 18-FDG incorporation in the lungs, a correlate of granulomatous TB disease, was lowered after D1MT+cART treatment. Since *M. tuberculosis*/SIV–coinfected RMs exhibited nonreactivator-like survival characteristics after early cART treatment (Figure 1A), and since TB disease was not detected by PET/CT in nonreactivators (Figure 1, B–L), we then compared the clinical progression in the 2 cART groups (D1MT+cART and cART) solely with untreated *M. tuberculosis*/SIV–coinfected reactivators.

### Clinical attributes of M. tuberculosis/SIV–coinfected RMs with D1MT+cART.

As in our previous reports, we used serum C-reactive protein (CRP) levels, weight loss, and serum albumin/globulin (A/G) ratios, as clinical correlates of TB reactivation in these groups. While untreated RMs progressively exhibited high serum CRP levels, those concurrently treated with D1MT+cART remained low, close to baseline, and comparable with the cART group and SIV mono-infected RMs (Figure 1M). D1MT+cART RMs also maintained their body weight for the duration of the protocol, akin to cART and SIV groups, while untreated RMs exhibited progressive weight loss after SIV coinfection (Figure 1N). We then measured other clinical attributes in RMs that have been shown to correlate with TB progression. No substantial changes in serum A/G ratios (Figure 1O) were observed among SIV monoinfected or *M. tuberculosis*/SIV–coinfected untreated, cART-treated, and D1MT+cART-treated RMs. The untreated group had lower A/G ratios at the endpoint relative to the 2 cART groups, indicative of more globulin in the serum due to the greater progression of *M. tuberculosis* infection in untreated animals. The serum A/G ratio was least affected in the SIV monoinfected RMs (Figure 1O). These results indicate that SIV monoinfection does not result in clinical disease like elevated serum CRP levels, reduced serum A/G ratios, or weight loss, all of which are clinical attributes of progressive *M. tuberculosis*/SIV coinfection. These clinical results show that concurrent administration of D1MT with cART is safe and does not promote inflammation or disease signatures associated with *M. tuberculosis* or SIV infections.

### IDO activity/expression.

**Figure 2:**
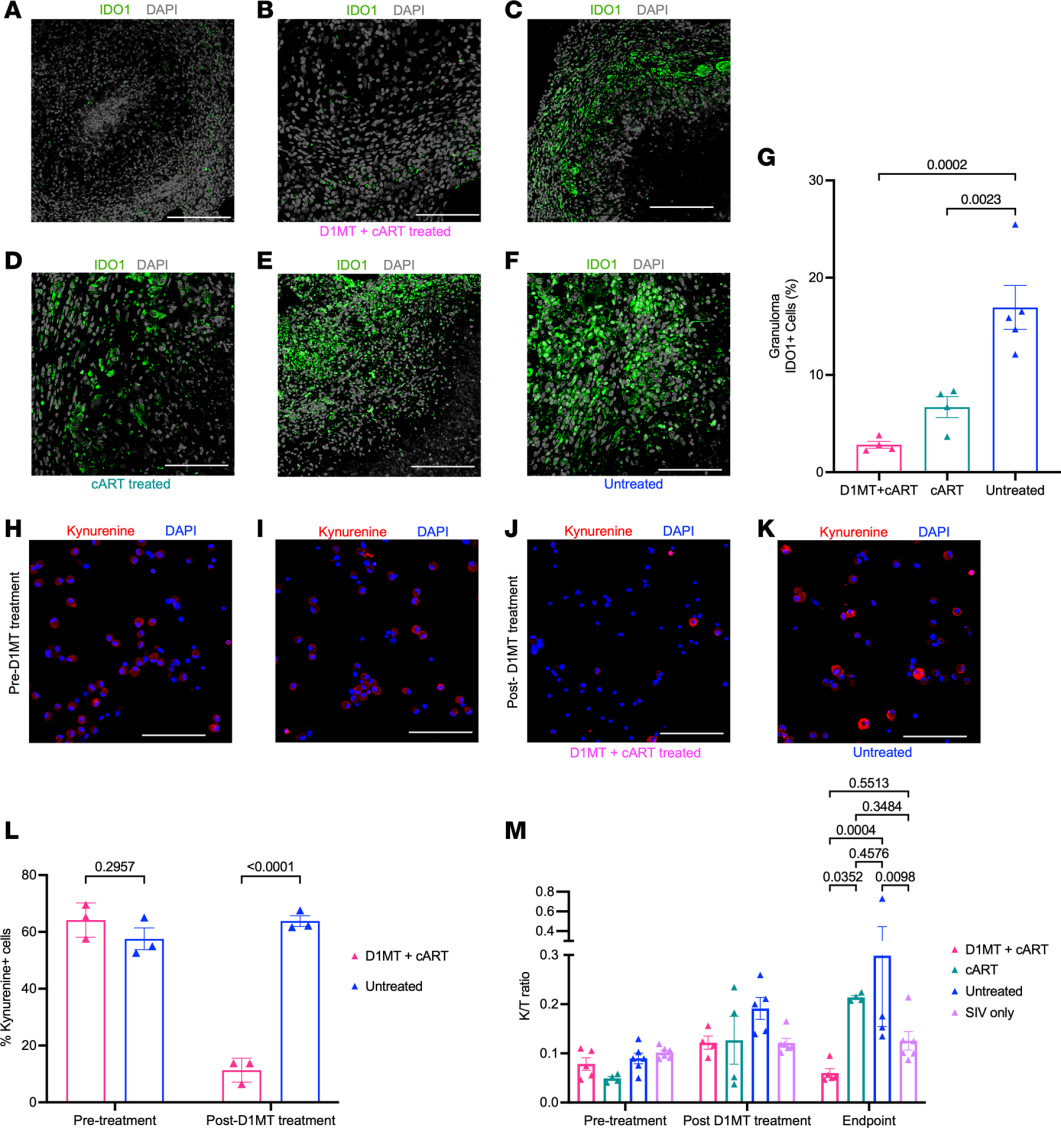
IDO activity/expression in D1MT+cART-treated relative to cART-treated *M. tuberculosis*/SIV–coinfected untreated and SIV monoinfected RMs. (**A**–**F**) Lung sections were stained for IDO1 and DAPI. The representative confocal images of the lung granulomas of the D1MT+cART-treated (**A** and **B**), cART-treated (**C** and **D**), and untreated (**E** and **F**) macaques showing IDO1 (green) and nuclei (gray) captured at ×10 (**A**, **C**, and **E**) and ×20 (**B**, **D**, and **F**) magnification. The whole tissue images were subsequently quantified using HALO software for IDO expression. Graph shows IDO1 expression in granulomas of D1MT+cART-treated, cART-treated, and untreated macaques (**G**). Immunofluorescence was also performed on BAL single-cell suspension collected on a slide surface using cytospin at wk11 (before D1MT treatment) and wk15 after infection (at the end of D1MT treatment) with Kyn antibody and DAPI. Representative confocal images (×20 magnification) showing kynurenine (red) and nucleus (blue) in BAL cells at pretreatment time point — i.e., wk11 (**H** and **I**) — and at the end of D1MT treatment — i.e., wk15 (**J** and **K**) — in the untreated and D1MT+cART groups. Twelve random fields/animal in each group were and quantified using ImageJ (Fiji) software. (**L**) Graph depicting the percentages of Kyn^+^ cells present in BAL pre- and post-D1MT treatment. (**M**) K/T ratios for D1MT+cART-treated**,** cART-treated, untreated, and SIV-only infected RMs at pretreatment, post-D1MT treatment, and endpoint. Scale bars: 200 μm (×10 magnification; **A**, **C**, and **E**), 100 μm (×20 magnification; **B**, **D**, **F**, and **H–K**). *P* values are indicated above the plots as obtained from 1-way ANOVA (**G**) and 2-way ANOVA (**L** and **M**). Data are represented as mean ± SEM.

The expression of IDO is highly induced in the lungs of NHPs during *M. tuberculosis* infection and *M. tuberculosis*/SIV coinfection (3, 4, 14, 17, 18, 20, 22, 24). We have earlier demonstrated that daily treatment with D1MT for 4 weeks is sufficient to inhibit in vivo IDO enzymatic activity in the lungs and reduces *M. tuberculosis*–induced expression of IDO transcript and protein (17, 19). We therefore studied if the inclusion of same daily D1MT treatment concurrent with cART could also similarly inhibit IDO activity and expression in a *M. tuberculosis*/SIV coinfection setting. For this purpose, we compared the effect of concurrent D1MT+cART treatment in *M. tuberculosis*/SIV–coinfected RMs with *M. tuberculosis*/SIV–coinfected/cART–treated RMs alone from historical data (2) and *M. tuberculosis*/SIV–coinfected RMs with either a clear reactivation or nonreactivation phenotype (from this study as well as from ref. 3), as shown in the experimental design (Supplemental Figure 1). The IDO levels were highly increased in the lungs of untreated RMs, with relatively lesser IDO levels in cART group, but there was significantly reduced IDO expression in D1MT+cART RM lung granulomas (Figure 2, A–G). Levels of kynurenine (Kyn), the product of direct IDO activity, were significantly decreased at the end of D1MT treatment, relative to before treatment initiation, as measured by immunofluorescence on longitudinally collected bronchoalveolar lavage (BAL) of the control and D1MT+cART RMs (Figure 2, H–L). Finally, we measured IDO enzymatic activity in the BAL of *M. tuberculosis*/SIV–coinfected, untreated RMs and compared it with D1MT+cART, cART, or SIV mono-infected animals (Figure 2M). This was important to study since IDO levels have also been reported to be increased in RMs by SIV monoinfection (25). We conducted quantitative ELISAs for the IDO substrate, Trp, and the product of IDO activity, Kyn, and expressed these results as Kyn/Trp (K/T) ratios (Figure 2M), as described earlier (17). K/T ratios in the BAL of untreated RMs were significantly higher than SIV monoinfected and D1MT+cART-treated, but not cART-treated, animals, although the latter group also exhibited a statistically nonsignificant decline in values (Figure 2M). These results clearly show that (a) SIV monoinfection does not induce IDO activity to the levels induced by *M. tuberculosis*/SIV coinfection; (b) cART treatment reduces IDO activity, due to control of TB reactivation; and (c) inclusion of D1MT concurrently with cART further significantly reduces IDO activity (Figure 2M). Overall, our results show that treatment with D1MT not only significantly inhibits IDO activity (Figure 2, H–M) but also affects IDO expression at the protein level (Figure 2, A–G, and M) in the setting of SIV coinfection and cART.

### Bacterial burdens in D1MT+cART-treated RMs relative to other groups.

**Figure 3:**
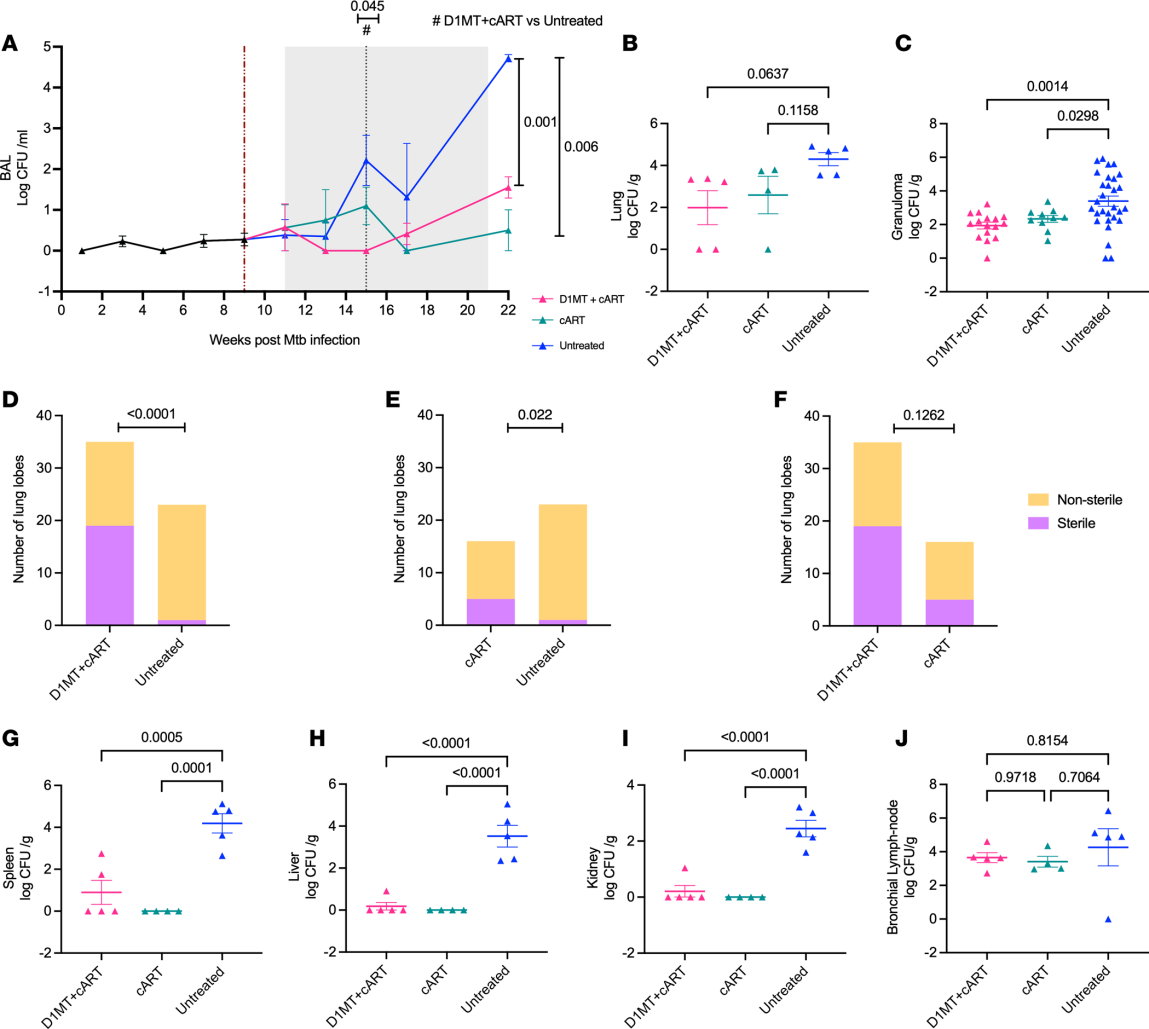
Bacterial burdens in D1MT+cART-treated RMs relative to other groups of coinfected RMs. *M. tuberculosis* burdens were assessed in BAL collected biweekly and tissues at endpoint. (**A**) Graph shows Log *M. tuberculosis* CFU/mL of BAL in the 3 study groups at different time points over the course ranging from preinfection to the end of treatment. Burgundy dotted line marks SIV infection, gray area represents the treatment phase, and black dotted line represents the end of D1MT treatment. (**B** and **C**) Graphical representations of Log_10_ CFU/gram of lung (**B**) and Log_10_ CFU/gram of granulomas (**C**) obtained from the lungs. (**D**–**F**) Individual lung lobes obtained from each RM were assessed for sterility using Fischer’s sterility test to determine the sterile and nonsterile lung lobes between D1MT+cART and untreated (**D**), cART and untreated (**E**), and D1MT+cART and cART (**F**). (**G**–**J**) Log CFU/gram of spleen (**G**), liver (**H**), kidney (**I**), and BrLN (**J**) obtained at necropsy. *P* values are indicated above the plots as obtained from 2-way ANOVA (**A**); 1-way ANOVA (**B**, **C**, and **H–J**); contingency χ^2^ (and Fisher’s exact) test (**D**–**F**). Data are represented as mean ± SEM.

We next studied *M. tuberculosis* burdens in various compartments of D1MT+cART RMs and compared these with cART-treated and untreated animals. Longitudinal BAL *M. tuberculosis* burdens in the D1MT+cART group remained close to baseline after SIV infection and for the duration of the treatment protocol and were indistinguishable from the cART-only group (Figure 3A). The untreated RMs exhibited progressively increasing BAL *M. tuberculosis* CFUs, such that, at the end of the study, these differences were statistically significant as compared with the 2 cART groups (Figure 3A). In lungs, the endpoint culturable *M. tuberculosis* CFUs for the D1MT+cART RMs were at a mean value of 1.99 logs/g, indistinguishable from the cART (2.59 logs/g) and the NR (1.74 logs/g) groups (Figure 3B). In contrast, the lung CFUs for the R group (a combination of RMs from this study and historical controls) were much higher (4.3 logs/g), which was significantly different from D1MT+cART group (Figure 3B). We also studied CFUs in individual lung granulomas isolated from these 4 groups of RMs. There was no statistical difference in the *M. tuberculosis* CFUs in granulomas between the D1MT+cART- and cART-treated groups, while the levels were significantly lower in the D1MT+cART (1.94 logs/g) and cART (2.33 logs/g) groups, relative to the R group (3.39 logs/g) (Figure 3C). Since we can measure lung *M. tuberculosis* CFU in samples collected from the individual lobes of each RM, we next studied if differences existed in the number of lung lobes that were sterile (no detectable culturable *M. tuberculosis*), versus nonsterile (detectable culturable *M. tuberculosis*) using Fisher’s test by comparing the untreated group to each of the other groups individually (Figure 3, D–F). About 55% of the lobes from RMs in the D1MT+cART group were sterile, compared with untreated animals (<5%) (Figure 3D). Furthermore, >30% of the lung lobes in the ART group were sterile, and these values were also significantly different when compared with the untreated group (Figure 3E). We also directly compared the presence of sterile lobes between the D1MT+cART and the cART groups, and while this difference was not statistically significant, almost twice the number of lobes obtained from the RMs of the former group were sterile compared with the latter (Figure 3F). Since SIV coinfection of *M. tuberculosis*–infected RMs leads to increased dissemination of bacilli in extrathoracic tissues (21, 22), we studied bacterial burdens in spleen (Figure 3G), liver (Figure 3H), and kidney (Figure 3I). Bacterial burdens in each of these 3 tissues of the D1MT+cART, cART, and NR groups were indistinguishable from each other, and much lower than CFUs from the R group (Figure 3, G–I). These results clearly show that concurrent D1MT treatment does not enhance dissemination of *M. tuberculosis* to extrathoracic tissues. *M. tuberculosis* CFUs in lung-draining bronchial lymph nodes (BrLN) were, however, comparable in all groups (Figure 3J), indicating that infection at this site is not governed by SIV-induced reactivation in NHPs. Together, our results show that the inclusion of D1MT is safe in the setting of cART treatment of *M. tuberculosis*/SIV coinfection and does not promote reactivation or increased *M. tuberculosis* replication.

### D1MT+cART treatment does not increase lung pathology.

**Figure 4:**
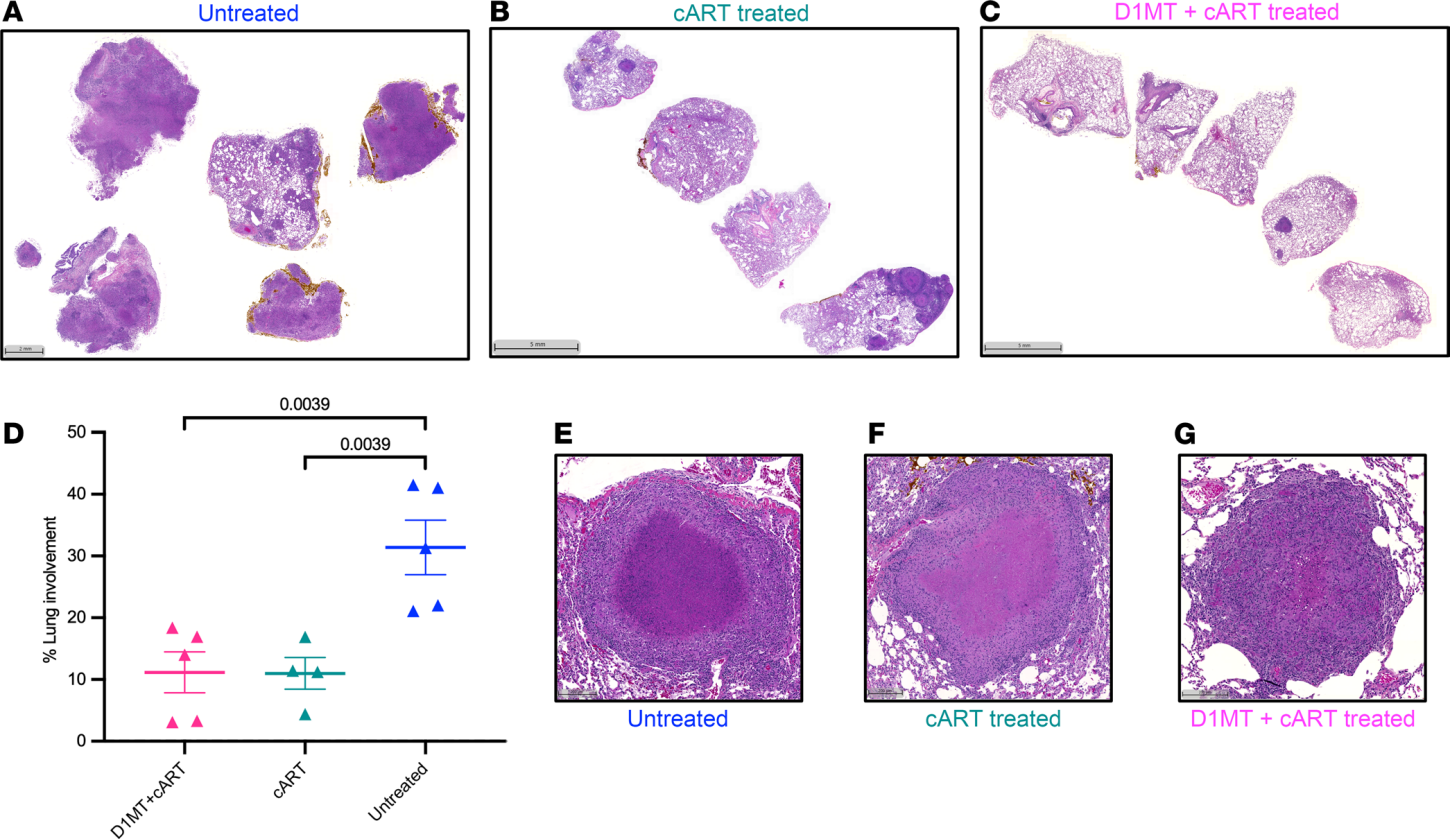
D1MT+ART treatment does not increase lung pathology. H&E staining was performed on stereologically representative lung tissues obtained at endpoint. (**A**–**C**) Shown are the representative images of H&E staining of lung from an untreated (**A**), cART-treated (**B**), and D1MT+cART-treated (**C**) RM. Scale bars: 2 mm (**A**) and 5 mm (**B** and **C**). These H&E-stained lung sections were used to quantify the lung area involved in inflammation or granulomatous lesions using HALO software. (**D**) The graph depicts the percentage lung involvement among the 3 groups of RMs. (**E**–**G**) Shown is the morphology of representative individual granuloma obtained from untreated (**E**), cART-only–treated (**F**), and D1MT+cART-treated (**G**) RMs. *P* values are indicated above the plots as obtained from 1-way ANOVA. Data are represented as mean ± SEM.

We assessed if IDO inhibition concurrent with cART caused enhanced tissue pathology. Gross and histopathological features of TB lung pathology and granuloma formation are shown in the lung in a representative RM from the untreated (Figure 4A), cART (Figure 4B), and D1MT+cART (Figure 4C) groups. Expectedly, widespread granulomatous pathology was observed in the lungs of untreated RMs, while TB pathology was found to be significantly reduced in both the cART groups. Morphometric measurement of lung pathology exhibited indistinguishable differences between the D1MT+cART (mean, ~11%) and cART groups (mean, ~11%), both of which were significantly lower than the lung pathology measured for the untreated group (mean, ~32%) (Figure 4D). The granulomas in D1MT+cART RMs generally appeared to be smaller in size and less necrotic (Figure 4G) as compared with the untreated group (Figure 4E) as well as the cART group (Figure 4F), although this was not quantified. These results support the clinical and microbiological results presented in Figure 1 and Figure 2 and clearly show that treatment with D1MT is completely safe in NHPs in the setting of *M. tuberculosis* infection and SIV coinfection, does not promote TB pathology, and could be administered in humans adjunctive to cART. Based on our results, it is possible that the inclusion of D1MT to cART prevents new granuloma formation after SIV coinfection, since D1MT-mediated IDO inhibition has been shown to enhance lung T cell responses and increase *M. tuberculosis* clearance.

### D1MT+cART controls viral replication as effectively as cART.

**Figure 5:**
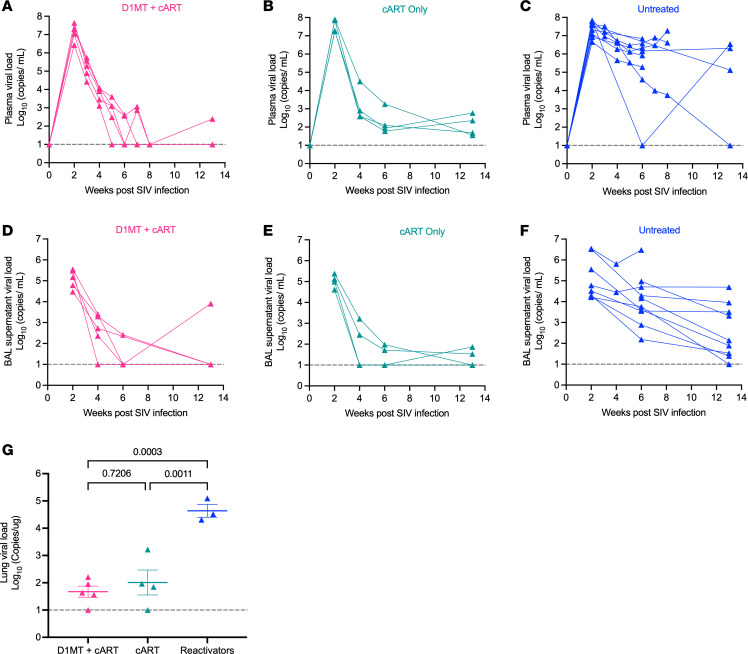
D1MT+cART controls viral replication as effectively as cART. (**A**–**C**) SIV titer in plasma of D1MT+cART-treated (**A**), cART-only–treated (**B**), and untreated controls (**C**) at different time points after SIV infection. (**D**–**F**) Viral load in BAL supernatant of D1MT+cART-treated (**D**), cART-only–treated (**E**), and untreated controls (**F**). Graph depicting viral load determined in the lungs from the RMs of the 4 groups (**G**). *P* values are indicated above the plot as obtained from 1-way ANOVA (**G**). Data are represented as mean ± SEM.

We compared SIV viremia in the peripheral and lung compartments of D1MT+cART- and cART-treated RMs and found no differences between the 2 groups. cART was initiated at week 11 after *M. tuberculosis* (2 weeks after SIV) (2), and in D1MT+cART- and cART-treated RMs, plasma SIV titers were lowered immediately from peak values of 7–8 logs (at 2 weeks after SIV), respectively, to ~3 logs by 4weeks after SIV — i.e., after 2 weeks of cART (Figure 5, A and B). There were no differences in the 2 groups, although most RMs in the D1MT+cART group had lower plasma SIV burdens at endpoint, such that detectable viral loads were present in only 1 of 5 animals in this group compared with 4 of 4 RMs in the cART group. In comparison, untreated animals had significantly higher SIV burdens at 4 weeks after SIV (Figure 5C) with detectable viral loads being present in 9 of 10 RMs. We also measured viral loads in the BAL longitudinally, and the 2 cART-treated groups of RMs were again indistinguishable from each other, with progressively lowering SIV burdens after cART treatment and with 1 of 5 and 2 of 4 RMs with detectable viral load at endpoint, respectively. On the other hand, higher viral loads were present in the BAL of untreated animals (9 of 10 with detectable viral load at endpoint) during the treatment phase, regardless of their reactivation status (Figure 5, D–F). Finally, at the endpoint, we measured SIV viral loads in the lung homogenates of all D1MT+cART- and cART-treated RMs and 6 (3 reactivators and 3 nonreactivators) untreated animals and again found that, while viral loads were detectable in each of the samples, the inclusion of D1MT to the cART regime concurrently had no effect on the lung SIV burden (Figure 5G). In contrast, untreated reactivators expectedly had statistically higher SIV titers in the lung homogenates than each of the other groups. Inclusion of D1MT to inhibit IDO signaling did not prevent cART-mediated clearance of SIV from the peripheral blood or the lung compartment during *M. tuberculosis*/SIV coinfection.

### Immune responses generated after D1MT+cART treatment.

**Figure 6:**
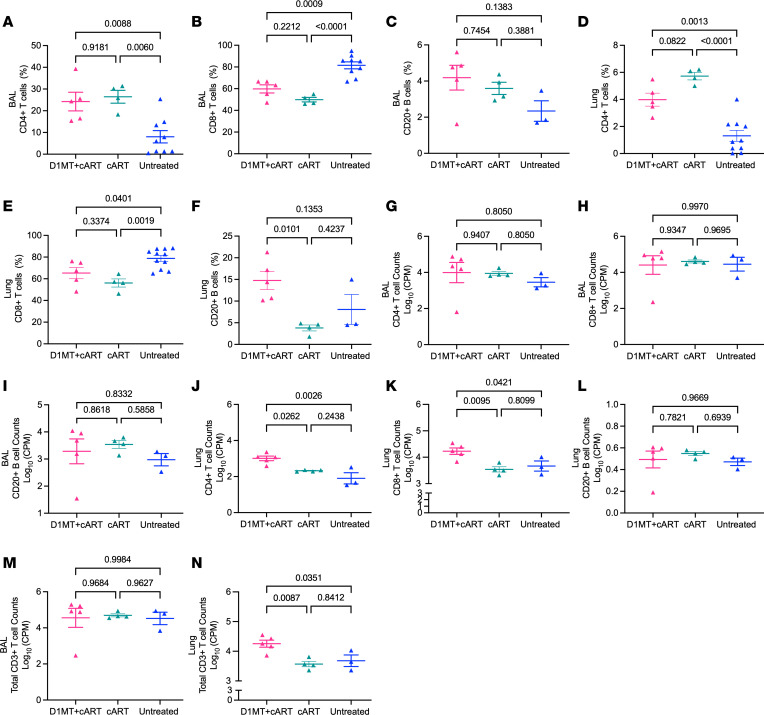
Immune responses generated after D1MT+cART treatment. (**A**–**F**) CD4^+^ and CD8^+^ T cell and CD20^+^ B cell frequencies in BAL (**A**–**C**) and lung (**D**–**F**) at endpoint, as determined by T cell flow cytometry panels. (**G** and **J**) Absolute log CD4^+^ T cell CPM in BAL (**G**) and lung (**J**). (**H** and **K**) Absolute log CD8^+^ T cell CPM in BAL (**H**) and lung (**K**). (**I** and **L**) Absolute log CD20^+^ B cell CPM in BAL (**I**) and lung (**L**). (**M** and **N**) Graph showing log CD3^+^ T cell CPM in BAL (**M**) and lung (**N**). *P* values are indicated above the plot as obtained from 1-way ANOVA (**A**–**N**). Data are represented as mean ± SEM.

We studied T cell dynamics in the BAL obtained from 3 groups of RMs: D1MT+cART, cART, and untreated (flow gating strategy is described in Supplemental Figure 2). T cell dynamics were also studied in the peripheral blood, where they were indistinguishable between groups. Higher frequencies of *M. tuberculosis*–specific T cell responses are known to be present in BAL, relative to blood (26). Finally, most phenotypes were also studied in endpoint lung tissue–derived cells. As expected, RMs of both the cART groups demonstrated a significant reconstitution (*P* < 0.01) of CD4^+^ T cell frequency in the endpoint BAL relative to untreated animals (mean, 8.03%), though the frequencies (24.24%, D1MT+cART; 26.45%, cART) were statistically indistinguishable for the 2 treatment groups (Figure 6A). Accordingly, a significant (*P* < 0.001) reduction in the frequency of CD8^+^ T cells was observed in the BAL of the 2 cART-treated RM groups (mean, 59.78% for D1MT+cART and 49.88% for cART) relative to the untreated group (mean, 81.51%) (Figure 6B). While more CD8^+^ T cells were recruited to the endpoint BAL of D1MT+cART than the cART animals, the difference in their frequency between the 2 cART groups was not significant. Interestingly, we also observed an increase in the frequency of (CD20^+^) B cells in the BAL of both cART groups relative to untreated animals, which was significantly higher in D1MT+cART relative to the cART group (Figure 6C). Reconstitution of CD4^+^ T cells was also observed in lung tissue at endpoint in the 2 cART groups, with each exhibiting a highly significant increase (mean, 3.99% and 5.72% for D1MT+cART and cART groups, respectively), relative to untreated RMs (mean, 1.30%) (Figure 6D). The differences between the 2 cART groups were not statistically significant. Again, a concomitant statistically significant decline in the CD8^+^ T cell frequency was observed in the endpoint lungs of D1MT+cART (mean, 65.26%) and cART-treated (mean, 56.10%) RMs relative to untreated controls (mean, 78.83%) (Figure 6E). The frequency of B cells was significantly higher in the lungs of the D1MT+cART (mean, ~15%), relative to untreated controls (mean, ~8%) but also cART group (mean, ~4%) (Figure 6F). A comparable increase in the CD4^+^ T cell frequencies was observed in other tissues — e.g., spleen (Supplemental Figure 3A) and BrLN (Supplemental Figure 3C) — which were statistically indistinguishable between the D1MT+cART and the cART groups. Similarly, the decreased frequencies of CD8^+^ T cells were also not statistically dissimilar in the 2 cART groups relative to untreated RMs, in spleen and BrLN (Supplemental Figure 3, B and D). While a similar result was obtained for the cART-only–treated animals longitudinally, the frequency (Supplemental Figure 4A) as well as the absolute magnitude (Supplemental Figure 4, B and C) of the CD4^+^ T cell reconstitution in the BAL improved with the addition of D1MT.

Reconstitution of CD4^+^ T cells in the BAL was also measured by absolute cell counts in flow cytometry. Greater numbers of CD4^+^ T cells were present in the BAL of the 2 cART groups at endpoint relative to the untreated group (Figure 6G and Supplemental Figure 4G). A mean of 4 log CD4^+^ T cells per million (CPM) or 33.7 × 10^3^ CPM were present in the BAL of D1MT+cART-treated group, while the mean for the cART-only group was 3.9 log CPM or 9.7 × 10^3^ CPM — i.e., ~3.5-fold lower than the D1MT group (Figure 6G and Supplemental Figure 4G). While the difference between the 2 cART groups was not significant, given the heterogeneity evident in the D1MT+cART group, clearly more total CD4^+^ T cells were recruited to the BAL by combined cART and D1MT treatment. Interestingly, the absolute numbers of CD8^+^ T cells also increased in the BAL of D1MT-treated RMs (70.7 × 10^3^ CPM), relative to both untreated (47.9 × 10^3^ CPM; ~1.5-fold lower than the D1MT group) and the cART-treated (42.5 × 10^3^ CPM; ~1.66-fold lower than the D1MT group) groups (Figure 6H and Supplemental Figure 4H). While the absolute numbers of B cells increased after cART treatment, the effects were not statistically significant (Figure 6I and Supplemental Figure 4, I–J). Increased absolute recruitment of CD4^+^ and CD8^+^ T cells to the lung after D1MT treatment were also observed at the endpoint. The absolute counts of CD4^+^ T cells in the lungs of D1MT+cART group (3 log CPM or 1.2 × 10^3^ CPM) were significantly higher, relative to not only the untreated group (1.9 log CPM or 0.14 × 10^3^ CPM; ~8.6-fold lower than the D1MT group) but also the cART-only (2.3 log CPM or 0.2 × 10^3^ CPM; 6-fold lower than the D1MT group) treatment group (Figure 6J). The counts of CD8^+^ T cells were also significantly higher in the D1MT+cART group (4.2 log CPM or 19.4 × 10^3^ CPM) relative to both the cART (3.5 log CPM or 3.7 × 10^3^ CPM; ~ 5.25-fold lower than the D1MT group) and the untreated (3.7 log CPM or 5.6 × 10^3^ CPM; ~ 3.5-fold lower than the D1MT group) groups (Figure 6K). Since the absolute counts of both CD4^+^ and CD8^+^ T cells were increased in the lungs of D1MT+cART-treated RMs, we studied if an increase was also observed in the total αβ T cell fraction (CD4^+^ and CD8^+^ T cells). Higher absolute counts of total T cells were indeed present in BAL (Figure 6L) and lungs (Figure 6M) of the D1MT+cART RMs at the endpoint. BAL contained higher absolute numbers of total T cells in D1MT+cART (4.6 log CPM or 104.4 × 10^3^ CPM) relative to cART-only (4.5 log CPM or 52.2 × 10^3^ CPM; 2-fold lower than the D1MT group) and untreated groups (4.5 log CPM or 52.1 × 10^3^ CPM; ~2-fold lower than the D1MT group), but these differences were not significant (Figure 6N). These counts were, however, significantly higher in the lungs of D1MT+cART group (4.3 log CPM or 20.6 × 10^3^ CPM) compared with cART (3.6 log CPM or 3.9 × 10^3^ CPM; ~5.3-fold lower than the D1MT group) and untreated (3.7 log CPM or 5.8 × 10^3^ CPM; ~3.55-fold lower than the D1MT group) groups. Our results clearly show that the increased frequency of CD8^+^ T cells in the BAL and lungs of D1MT+cART-treated RMs was not just due to the lower frequencies of CD4^+^ T cells by SIV coinfection, as the absolute number of both CD4^+^ and CD8^+^ T cells increased after D1MT+cART treatment. While the increased reconstitution of CD4^+^ T cells is expected because of the effect of cART on SIV burdens, the significantly increased influx of CD8^+^ T cells in the lungs of the D1MT+cART group is strongly indicative of the HDT potential of IDO blockade. Increased T cell recruitment to the lung compartment due to the addition of D1MT-treatment adjunctive and concurrently with cART initiation not only occurred at endpoint but also longitudinally, and the effects were apparent after 4 weeks of D1MT treatment (week 15). Thus, both CD4^+^ T cell frequencies (Supplemental Figure 4A) and absolute counts (Supplemental Figure 4B) were higher at week 15 and endpoint for the D1MT+cART group relative to untreated controls, in the current study. Concomitant with the increase in CD4^+^ T cell frequencies, those of CD8^+^ T cells were longitudinally reduced in BAL (Supplemental Figure 4D), but absolute counts increased at both week 15 (the conclusion of D1MT treatment) and endpoint (the conclusion of cART) (Supplemental Figure 4, E and F). That the immunomodulatory effect of IDO is, in part, mediated by its inhibition of CD8^+^ T cells is a well studied phenomenon in both animal models (27) and humans (28). Since we observed increased absolute counts of both CD4^+^ and CD8^+^ T cells in D1MT+cART group, these results show that the inclusion of D1MT and the resulting IDO inhibition has no adverse effect on the reconstitution of CD4^+^ T cell frequencies in the lung as well as the airways due to cART and instead promotes the increased recruitment of absolute numbers of both CD4^+^ and CD8^+^ T cells to the lungs. These results are consistent with the immunoregulatory function of IDO and the known effect of D1MT treatment in vivo.

While the reconstitution of CD4^+^ T cells was expected, due to cART, the increase in CD8^+^ T cell counts after D1MT+cART treatment indicated that IDO inhibition resulted in an overall improvement of the quality of immune responses. We therefore also studied B cell recruitment after D1MT+cART treatment. As shown in Figure 6, C, G, and I, the frequency and the count of B cells were increased in D1MT+cART treatment in BAL and lung. Using confocal microscopy, we validated this result (Supplemental Figure 5). In lung sections stained for CD20, a canonical B cell marker (Supplemental Figure 5, A–F), significantly more staining was observed for RMs in the D1MT+cART, relative to not only the untreated group but also the cART-only group (*P* = 0.0549) (Supplemental Figure 5G). Although IDO was initially thought to be important for the regulation of T cell responses, emerging evidence suggests that it plays an important role in B cell signaling as well (29). SIV/HIV infection is well known to drive inflammatory, dysregulated B cell responses (30). Thus, our results suggest that IDO inhibition not only improves T cell reconstitution to the lung compartment but also enhances productive B cell responses. We also studied if the recruitment of NK cells was increased by IDO inhibition and found that this was not the case. The frequency of NKG2a^+^CD3^–^CD20^–^CD14^–^CD66abce^–^ cells (NK cells) was slightly higher in the D1MT+cART group relative to other groups, but the difference was not statistically significant (Supplemental Figure 5, H–N).

**Figure 7:**
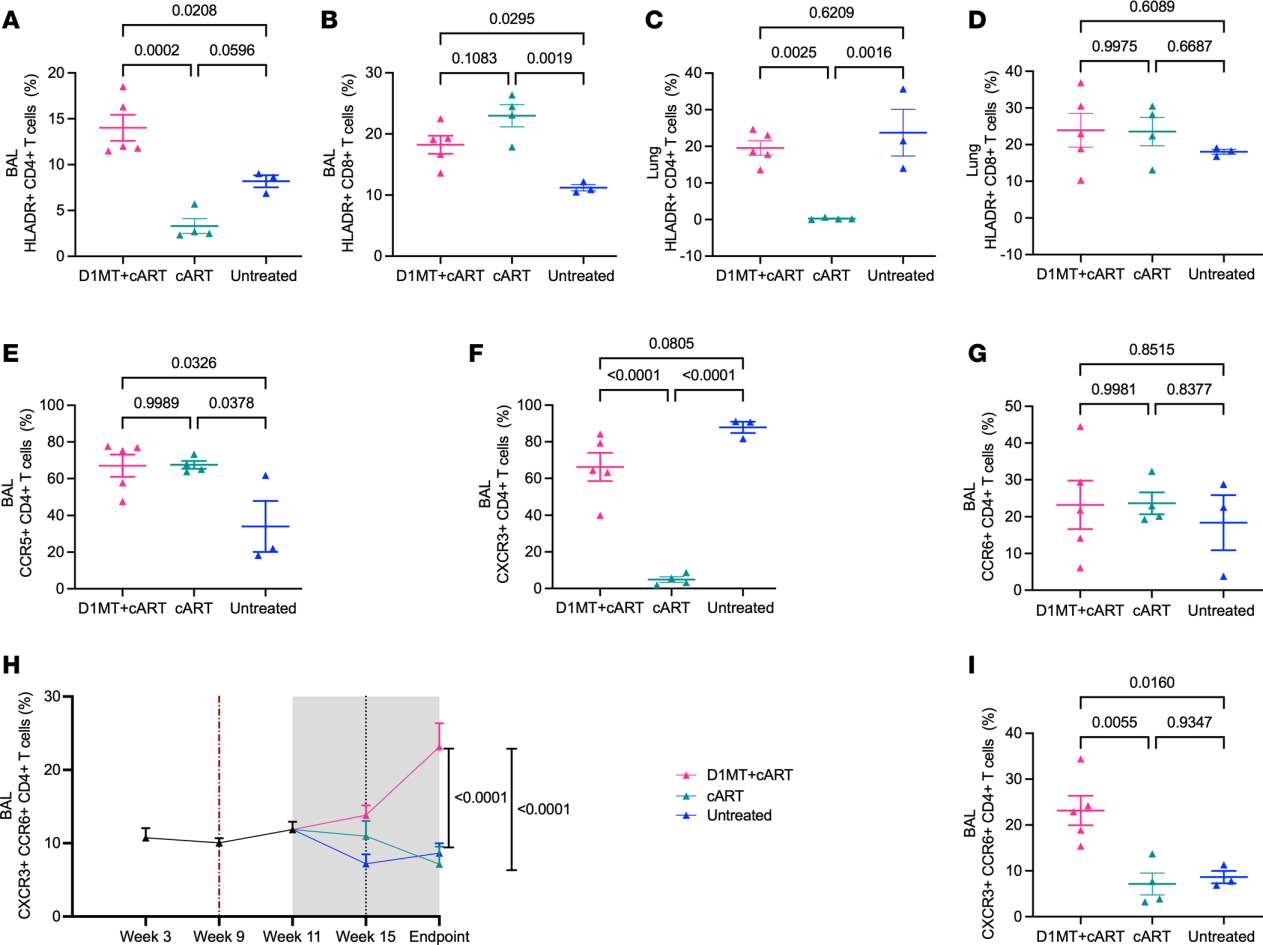
T cell phenotyping. (**A**–**D**) Graphs showing percentages of HLADR^+^CD4^+^ T cells in BAL (**A**) and lung (**C**), and percentages of HLADR^+^CD8^+^ T cells in BAL (**B**) and lung (**D**). (**E**–**G**) Graphs showing percentages of CCR5^+^, CXCR3^+^, and CCR6^+^CD4 T cells in BAL. (**H**) Percentages of CXCR3^+^CCR6^+^CD4^+^ T cells in BAL at various time points in D1MT+cART-treated, cART-treated, and untreated groups. (**I**) Graphical representation of CXCR3^+^CCR6^+^CD4^+^ T cell percentages in BAL at endpoint. *P* values are indicated above the plot as obtained from 1-way ANOVA (**A**–**G**, and **I**) and 2-way ANOVA (**H**). Data are represented as mean ± SEM.

Inclusion of D1MT concurrently with cART not only increased absolute T cell numbers in the BAL and lungs, but it also resulted in significantly increased activation and proliferation phenotype. Thus, significantly greater frequencies of HLA-DR^+^CD4^+^ T cells (mean, 14.02%) were present in the BAL of D1MT+cART group relative to the cART (mean, 3.31%) and the untreated (mean, 8.18%) groups (Figure 7A). A similar effect was observed for the HLA-DR^+^CD8^+^ T cell population, where significantly greater frequencies of HLA-DR^+^CD8^+^ T cells (mean, 18.24%) were present relative to the control group (mean, 11.20%) (Figure 7B). HLA-DR is a canonical marker of T cell activation. A similar result was obtained in the lung samples where statistically greater frequencies of HLA-DR^+^CD4^+^ (Figure 7C) T cell populations were present in the D1MT+cART group (mean, 19.56%) at endpoint, relative to the cART group (mean-0.26%). Our results show that the HLA-DR frequencies of the untreated *M. tuberculosis*/SIV–coinfected RMs were comparable with those of the D1MT+cART-treated animals and were significantly higher than the cART-only–treated animals, but the clinical status of the D1MT-treated (controlled *M. tuberculosis* infection) and untreated (TB reactivation) RMs was quite different (Figure 1 and Figure 2), as was the case in lung bacterial burdens (Figure 3). Our results, therefore, indicate that activation of T cells via HLA-DR by IDO inhibition — concurrent with cART, which restricts SIV viremia and controls TB reactivation, thus limiting *M. tuberculosis* antigenic burden — does not cause chronic immune activation observed in cART-naive *M. tuberculosis*/SIV–coinfected RMs. We and others have shown that the induction of IDO expression in granulomas from patients with TB associates with HLA-DR downregulation, which is consistent with immune evasion that disables antigen presentation to CD4^+^ T cells (8). Enhanced HLA-DR expression and T cell activation by IDO inhibition, in the absence of an ongoing infection, suggests the optimization of immune responses by D1MT treatment. Frequencies of HLA-DR^+^CD8^+^ T cells (Figure 7D) were also higher in lungs of D1MT+cART group, but differences were not significant. When HLA-DR frequencies of CD4^+^ and CD8^+^ T cells were measured longitudinally in BAL, between the D1MT+cART and the untreated control animals in the recent experiment (not in the historical controls for which these data were not collected), a significantly higher level was detected in the treatment group (Supplemental Figure 6, A and B), again underscoring that D1MT+cART treatment enhances T cell activation phenotype in the BAL. In these 2 groups we could also measure the frequency of CD4^+^ and CD8^+^ T cells expressing Ki67, a canonical marker of increased T cell proliferation, in BAL, and we found increased proliferative capacity in samples derived from the D1MT+cART rather than the untreated group (Supplemental Figure 6, C and D). The frequencies of Ki67^+^CD4^+^ (Supplemental Figure 6C) and Ki67^+^CD8^+^ (Supplemental Figure 6D) populations were also highly increased after D1MT treatment relative to other groups, although the effect was not statistically significant due to heterogeneity in data obtained from the low-dose infection model. Thus, in conclusion, including D1MT concurrently with cART did not interfere with the reconstitution of CD4^+^ T cells to the lung compartment and other tissues, and in fact, it enhanced the proliferative and activation status of T cell populations.

Chemokine receptors CCR5, CXCR3, and CCR6 are important in the delineation of T cell phenotypes in the lung (26) as well as in the context of coinfection (31). While both CCR5 and CXCR3 are markers of Th1 immunity, CCR6^+^ cells represent Th17 phenotype (26). We therefore used these markers to study T cell phenotypes in response to D1MT+cART and cARTtreatments in the lung compartment. The significant majority of CD4^+^ T cells reconstituted to the BAL of the 2 cART-treated groups were CCR5^+^ (Figure 7E and Supplemental Figure 6), results which are supported by previous studies (32). In lungs, at endpoint, the increase in the frequencies of CD4^+^CCR5^+^ cells in the 2 cART groups (mean, 29.96% and 33.23% for D1MT+cART and cART groups, respectively), relative to the untreated group (mean-24.47%) was not statistically significant (Supplemental Figure 7A). While CCR5^+^CD4^+^ T cells are a preferential host for SIV/HIV, we did not observe higher viral loads in D1MT+cART relative to the cART group of RMs (Figure 5, A, B, D, E, and G).

The frequency of CXCR3^+^CD4^+^ T cells was significantly increased in only the BAL of D1MT+cART-treated RMs, relative to cART-treated animals but not untreated RMs, mirroring the result obtained for HLA-DR^+^CD4^+^ T cells (Figure 7F and Supplemental Figure 7D). The frequency of CXCR3^+^CD4^+^ T cells (Th1) has been inversely correlated with *M. tuberculosis* burdens (26). Thus, both CXCR3 (Figure 7F) and HLA-DR (Figure 7, A and C) expression on CD4^+^ T cells was comparably increased after D1MT treatment. Despite this, no increase in SIV viremia was observed after D1MT treatment (Figure 5, A, D, and G). Increased frequencies of CXCR3^+^CD4^+^ T cells resulted in concomitantly lower CXCR3^+^CD8^+^ T cell frequencies in the BAL of the D1MT+cART group as compared with untreated RMs (Supplemental Figure 7E). Surprisingly, there were significantly lower CXCR3^+^CD4^+^ T cells in lungs of both treated groups as compared with the untreated group (Supplemental Figure 7B). These results suggest that while cART reduces inflammation and TB reactivation — thereby decreasing *M. tuberculosis* antigen levels in the lung, resulting in a decrease in the overall Th1 CD4^+^ T cell population — the concurrent addition of the IDO inhibitor D1MT to cART results in significantly increased frequencies of CXCR3^+^CD4^+^ T cells in the BAL (airways), but not the lung, via mechanisms we do not understand. Trafficking of immunocytes between the lumen (airways) and the parenchyma during TB and *M. tuberculosis*/SIV coinfection is a critical new area of research.

The frequency of CCR6^+^CD4^+^ T cells was unaffected across groups in BAL (Figure 7G) as well as lungs (Supplemental Figure 7C). However, the frequencies of CCR5^+^CD8^+^ T cells were not lowered by D1MT treatment (Supplemental Figure 7H). Our results suggest that the CD4^+^ T cells reconstituted to the lung compartment after cART are of the CCR5^+^ variety (Supplemental Figure 7G), and coadministration of D1MT further enhances their Th1 (CXCR3) phenotype. Th1 phenotype CD4^+^ T cells are primarily depleted from the lung granulomas of RMs coinfected with *M. tuberculosis*/SIV (31). Our results suggest that inclusion of a Th1 response potentiator such as D1MT can result in a more efficient restoration of these responses adjunctive to cART. Interestingly, while there were no differences in the levels of CCR6^+^ T cells after treatment, the frequency of CXCR3^+^CCR6^+^ (Th* or Th1Th17 phenotype)CD4^+^ T cells (26) was significantly increased in the BAL of D1MT+cART-treated RMs as compared with untreated (Figure 7, H and I) and cART-treated RMs (Figure 7I). This effect was specific to CD4^+^ and not CD8^+^ T cells; thus, the frequency of CXCR3^+^CCR6^+^CD8^+^ T cells was comparable in BAL of D1MT+cART and untreated groups (Supplemental Figure 7F). We have previously identified a strong correlation between these Th*CD4^+^ (but not CD8^+^) T cells and control of TB in RM lungs (26). IDO is the key regulator of the Treg/Th17 balance (33) via aryl hydrocarbon receptor signaling (34). These results suggest that IDO inhibition caused by D1MT treatment enhances Th17 and, particularly, Th* recruitment to the lungs. Overall, our study of the T cell phenotypes recruited to the lungs of untreated, cART-treated, or D1MT+cART-treated RMs suggests that, while cART increases immune reconstitution to the lung by controlling viral infection, D1MT improves the quality of these T cell responses by enhancing activation, proliferation, and Th1 and Th* phenotypes in the absence of chronic immune activation.

### M. tuberculosis specificity of the immune responses generated after D1MT+cART treatment.

**Figure 8:**
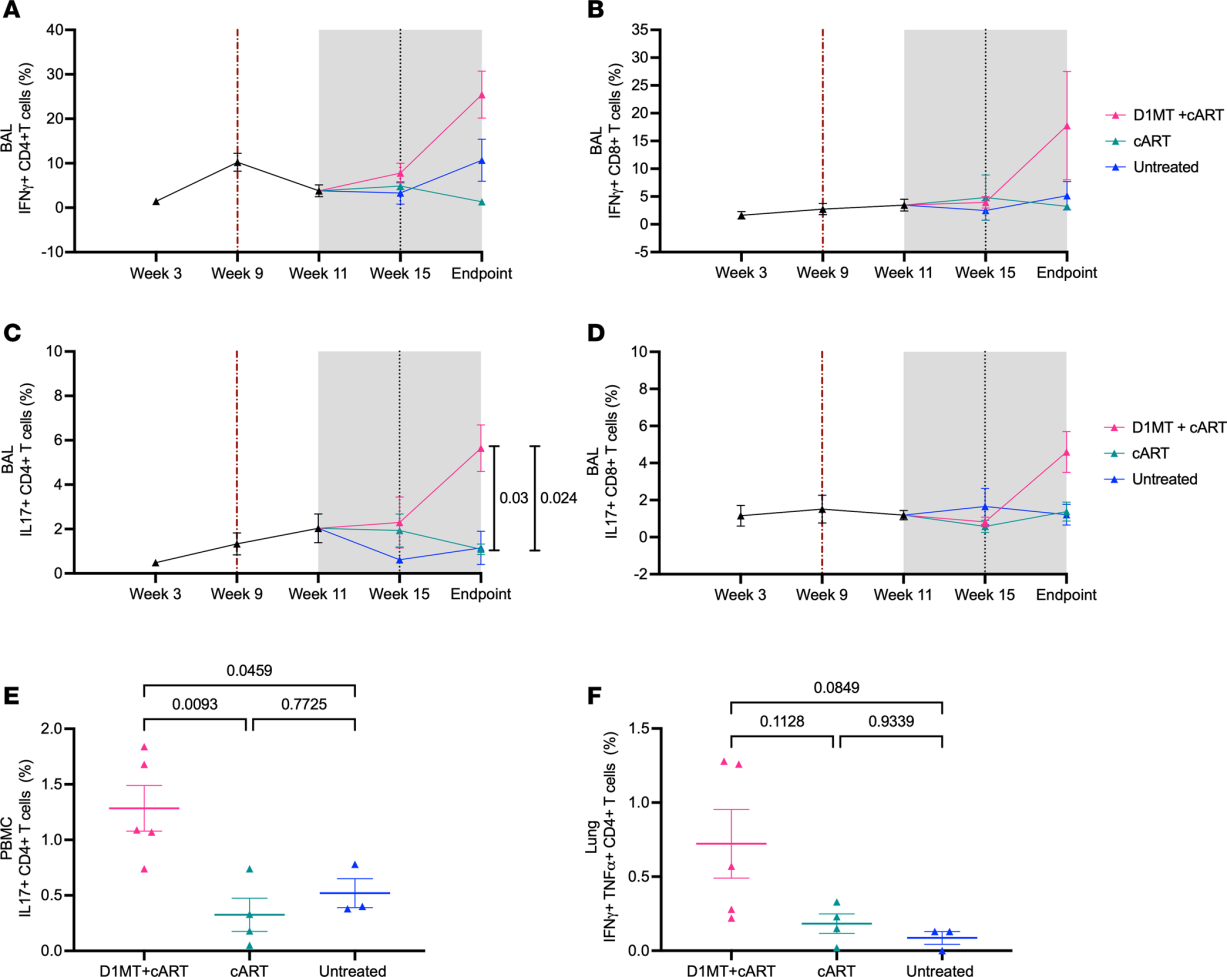
*M. tuberculosis* specificity of the immune responses generated after D1MT+cART treatment. (**A** and **B**) Graphical representations of *M. tuberculosis* CW-specific IFN-γ^+^CD4^+^ T cell (**A**) and IFN-γ^+^CD8^+^ T cell (**B**) percentages in BAL at various time points. (**C** and **D**) Graphs showing *M. tuberculosis* CW-specific IL-17^+^CD4^+^ T cell (**C**) and IL-17^+^CD8^+^ T cell (**D**) percentage in BAL. (**E** and **F**) Percentage of *M. tuberculosis* CW-specific IL-17^+^CD4^+^ T cells in PBMCs at endpoint (**E**) and percentage of *M. tuberculosis* CW-specific IFN-γ^+^TNF-α^+^CD4^+^ T cells in lung (**F**) of untreated, D1MT+cART, and cART groups. *P* values are indicated above the plot as obtained from 2-way ANOVA (**A**–**D**) and 1-way ANOVA (**E** and **F**). Data are represented as mean ± SEM.

To understand if the cellular immune responses induced during IDO inhibition in *M. tuberculosis*/SIV–coinfected, cART-treated RMs were *M. tuberculosis*–specific and polyfunctional, we subjected samples from BAL, lungs, and peripheral blood to antigen restimulation ex vivo and performed intracellular cytokine staining (ICCS). Significantly higher levels of antigen-specific (*M. tuberculosis* cell wall [CW]) IFN-γ–expressing CD4^+^ T cell responses were observed from the BAL of D1MT+cART-treated relative to cART-treated or untreated RMs (Figure 8A). A similar increase was also observed in the D1MT+cART group for *M. tuberculosis* CW-specific, IFN-γ–expressing CD8^+^ T cells (Figure 8B). However, no differences in IFN-γ–expressing CD4^+^ T cells was observed in lungs among the 3 groups (Supplemental Figure 8G). Comparable with the results obtained for IFN-γ, the levels of *M. tuberculosis* CW-specific, IL-17–expressing CD4^+^ (Figure 8C) and CD8^+^ (Figure 8D) T cells were also significantly higher in the BAL of D1MT+cART, relative to the other 2 groups. The levels of IL-17–expressing (Figure 8E) but not IFN-γ–expressing CD4^+^ T cells were also significantly higher in the PBMCs of D1MT+cART, relative to the other 2 groups. The levels of *M. tuberculosis* CW-specific, TNF-α–expressing CD4^+^ (Supplemental Figure 8, A and H) and CD8^+^ (Supplemental Figure 8B), and those of *M. tuberculosis* CW-specific, IFN-γ/TNF-α–expressing bifunctional CD4^+^ (Supplemental Figure 8C) and CD8^+^ (Supplemental Figure 8D) T cells, were also increased in the BAL and lungs of D1MT+cART-treated RMs, relative to the other 2 groups, although these differences were not significant (Figure 8F). Additionally, *M. tuberculosis* CW-specific, IFN-γ/TNF-α/IL-17–expressing polyfunctional CD4^+^ T as well as CD8^+^ T cells were higher in BAL of D1MT+cART-treated groups, whereas these frequencies were lower and comparable in cART and untreated groups (Supplemental Figure 8, E and F). These results show that the inclusion of D1MT to the cART regime in *M. tuberculosis/*SIV coinfection significantly increases the antigen-specific T cell response specific to proinflammatory Th1 and Th17 immunity, without increasing lung pathology (Figure 4); burdens of either *M. tuberculosis* (Figure 3) or SIV (Figure 5); or clinical markers of disease such as serum CRP or A/G ratio and 18-FDG incorporation in lung lesions (Figure 1).

### Dynamics of T cells and macrophages in the lung granulomas after D1MT+cART and cART.

**Figure 9:**
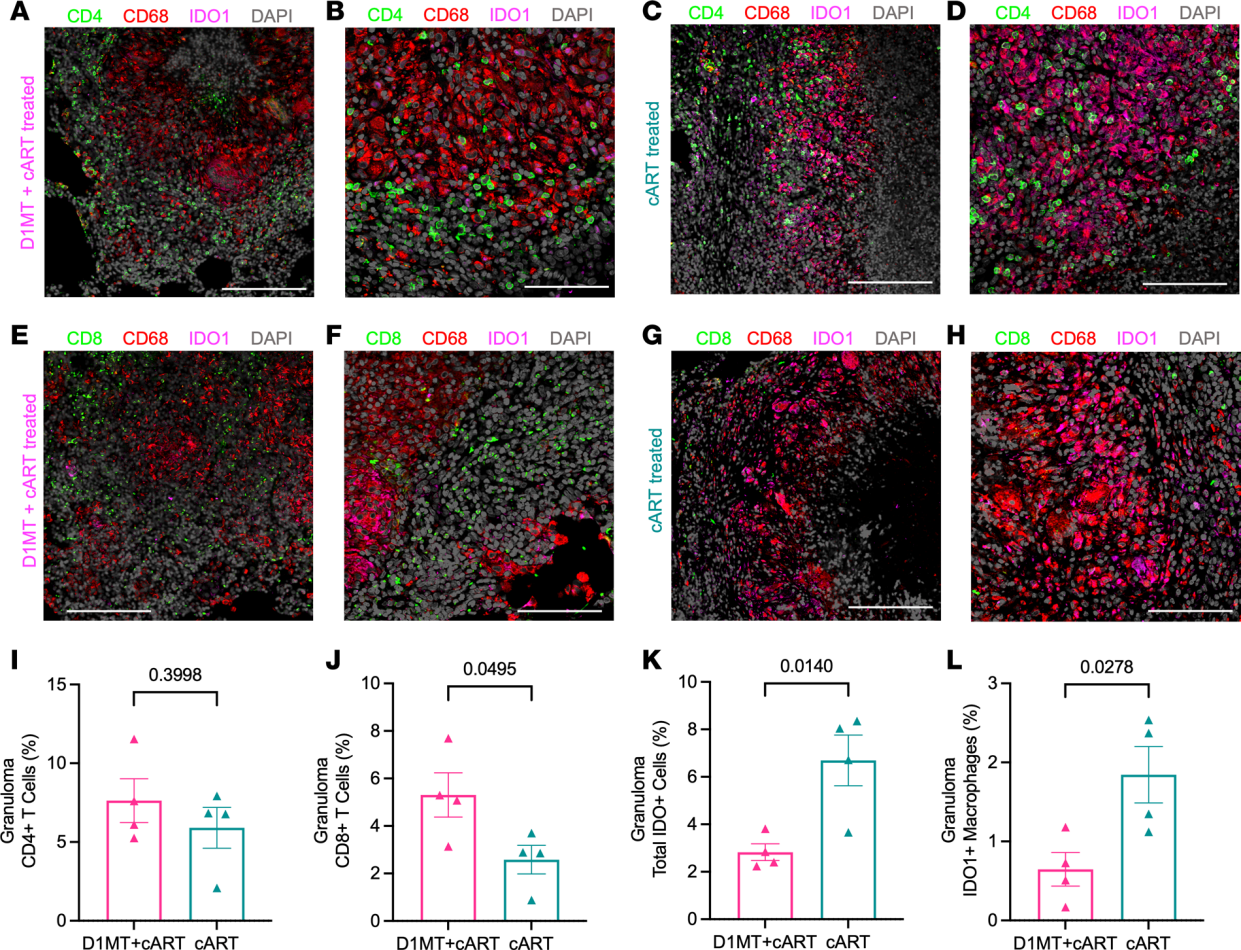
Dynamics of T cells and macrophages in the lung granulomas after D1MT+cART and cART treatment. (**A**–**H**) Lung sections obtained at necropsy of RMs from all the study groups were stained for CD4, CD8, CD68, and IDO1. The multilabel confocal images of the lung granulomas of the D1MT+cART- and cART-treated macaques depicting CD4/CD8 (green), CD68 (red), IDO1 (magenta), and nucleus (gray) captured at ×10 (**A**, **C**, **E**, and **G**) and ×20 (**B**, **D**, **F**, **H**) magnification. Representative images of CD4, CD68, and IDO1 in D1MT+cART RM are shown (**A** and **B**) as well as in cART-treated RM (**C** and **D**). Representative images of CD8, CD68, and IDO1 in D1MT+cART (**E** and **F**) and in cART (**G** and **H**) groups. These representative images were captured using Zeiss LSM-800 confocal microscope. The whole-tissue sections were scanned and quantified for CD4^+^ T cells, CD8^+^ T cells, total IDO1 expressing cells, and IDO1^+^ macrophages using HALO. (**I**–**L**) Graph showing the comparison of percentages of CD4^+^ T cells (**I**), CD8^+^ T cells (**J**), IDO1^+^ cells (**K**), and IDO1^+^ macrophages (**L**) in lung granuloma of D1MT+cART and cART-only group. Scale bars: 200 mm (×10 magnification; **A**, **C**, **E** and **G**) and 100 mm (×20 magnification; **B**, **D**, **F** and **H**). *P* values are indicated above the plots as obtained from unpaired *t* test (**I**–**L**). Data are represented as mean ± SEM.

We have previously demonstrated that the granulomas of *M. tuberculosis–*infected asymptomatic controllers is characterized by the presence of inducible bronchus associated lymphoid tissue (iBALT) (35, 36), which plays an important role in the induction of protective CD4^+^ T cell responses (37). *M. tuberculosis*/SIV–coinfected reactivator RMs are characterized by the presence of greater inflammation and depletion of iBALT (20, 22). Importantly, regions of the lung granulomas previously occupied by iBALT are instead populated by IDO^+^ macrophages, and cART does not reverse this (3). IDO is primarily expressed on recruited, inflammatory, interstitial macrophages (IMs) in the lungs (4). The massive recruitment and rapid turnover of IMs, rather than the levels of CD4^+^ T cells, is highly correlated with the *M. tuberculosis*/SIV coinfection reactivation phenotype (38). To study the effect of IDO inhibition on the dynamics of different T cell types in granulomas at endpoint, multilabel IHC was performed on lung sections (containing granulomas) obtained from D1MT+cART-treated and cART-only treated RMs for CD4, CD8, IDO1, and CD68 (pan macrophage marker). Representative images show stains for CD4/CD68/IDO1 (Figure 9, A–D) and CD8/CD68/IDO1 (Figure 9, E–H) in D1MT+cART-treated (Figure 9, A, B, E, and F) and cART-treated (Figure 9, C, D, G, and H) RM granulomas (Figure 9, A–H). We observed similar frequencies of CD4^+^ T cells (Figure 9I) but significantly higher frequencies of CD8^+^ T cells (Figure 9J) in the granulomas of D1MT+cART-treated as compared with cART-only–treated RMs. These results are consistent with our earlier observations in *M. tuberculosis* monoinfected RMs where a greater frequency of T cells could be detected in the lung lesions of D1MT-treated animals (17). Moreover, a significantly higher number of total IDO1-expressing cells (Figure 9K) and IDO1-expressing macrophages (Figure 9L) in the granulomas of the cART-only group were significantly higher as compared with D1MT+cART group. Two results are noteworthy. Interestingly, while T cell reconstitution to the lungs was a feature of both cART-treated groups, D1MT+cART animals were characterized by the greater recruitment of CD8^+^ T cells to the lung lesions. Our results suggest that the greater killing of *M. tuberculosis* during IDO inhibition could be the result of enhanced CD8^+^ T cell responses. These results also show that, while chronic immune activation is not eliminated by cART even when initiated at week 11 (2 weeks after SIV infection) (2), chemical inhibition of IDO in vivo results in a significant reduction of chronic immune activation due to SIV infection. As a result, the recruitment of inflammatory macrophages, which express IDO, to the lungs is significantly blocked.

### Measurement of chronic immune activation in RM lungs after D1MT+cART and cART.

Our results suggest that inclusion of D1MT, better controls chronic immune activation generated by SIV coinfection. We therefore studied this in depth. We have earlier shown that inflammation in the lungs during 

**Figure 10:**
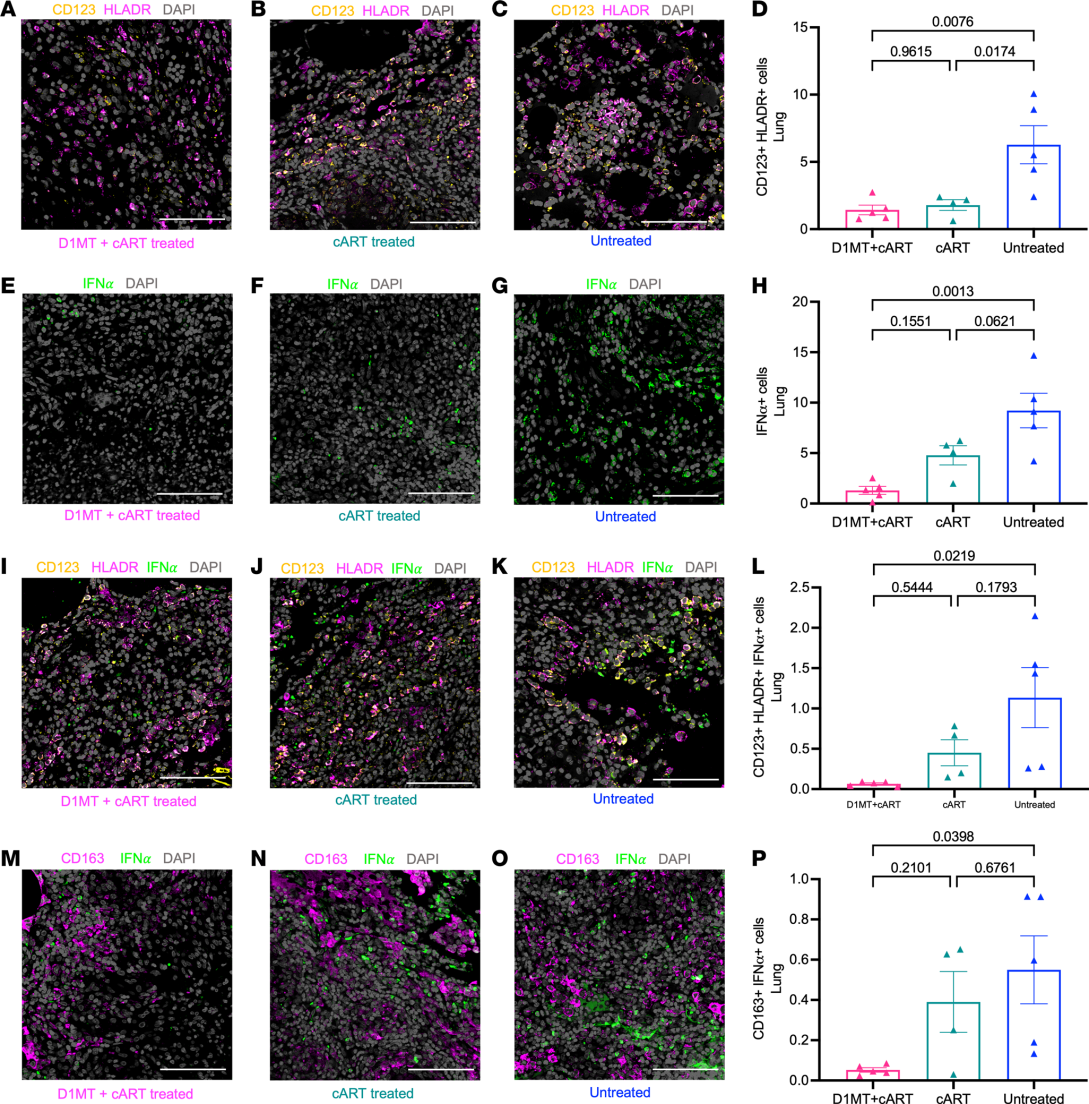
Measurement of chronic immune activation in the lung of treated and untreated groups. (**A**–**C**, **E**–**G**, **I**–**K**, and **M**–**O**) Lung sections were stained for CD123 and HLA-DR; IFN-α; CD123, HLA-DR, and IFN-α; and CD163 and IFN-α. The representative confocal images of the lung of the D1MT+cART-treated (**A**, **E**, **I**, and **M**), cART-treated (**B**, **F**, **J**, and **N**), and untreated (**C**, **G**, **K**, and **O**) macaques showing CD123 (yellow), HLA-DR (magenta), IFN-α (green), CD163 (magenta), and nuclei (gray) captured at ×20 magnification. (**D**, **H**, **L**, and **P**) Graph depicting percentages of pDCs (CD123^+^HLA-DR^+^), IFN-α^+^ cells, IFN-α^+^ pDCs, and CD163^+^ macrophages expressing IFN-α, in lungs of D1MT+cART-treated, cART-treated, and untreated macaques. Scale bars: 100 μm. *P* values are indicated above the plots as obtained from 1-way ANOVA (**D**, **H**, **L**, and **P**). Data are represented as mean ± SEM.

*M. tuberculosis* infection correlates with type I IFN signaling (4). Type I IFN is expressed on plasmacytoid DCs (pDCs). Using confocal microscopy, we therefore studied the recruitment of pDCs in the lungs of the study groups. Significantly fewer CD123^+^HLADR^+^ cells (pDCs) were present in the lungs of both cART-treated groups relative to untreated RMs (Figure 10, A–D). The frequency of IFN-α^+^ cells in the lungs of the D1MT+cART group, but not the cART-alone group, was also significantly lower than in untreated animals (Figure 10, E–H). Similar results were obtained with IFN-α^+^ pDC staining (Figure 10, I–L). Reduction of IFN-α^+^ pDCs in the D1MT+cART but not the cART group was statistically significant, relative to the untreated group. Type-I IFN signaling in pDCs induces the recruitment of IMs that express immunoregulators like IDO (4) and PD-L1 (8). We therefore studied the frequency of inflammatory IMs in the lungs. While cART treatment (~0.4%) failed to alter the frequency of these cells in the lungs relative to untreated RMs (~0.55%), D1MT+cART-treated (~0.05%) animals had significantly lower frequency relative to untreated RMs.

**Figure 11:**
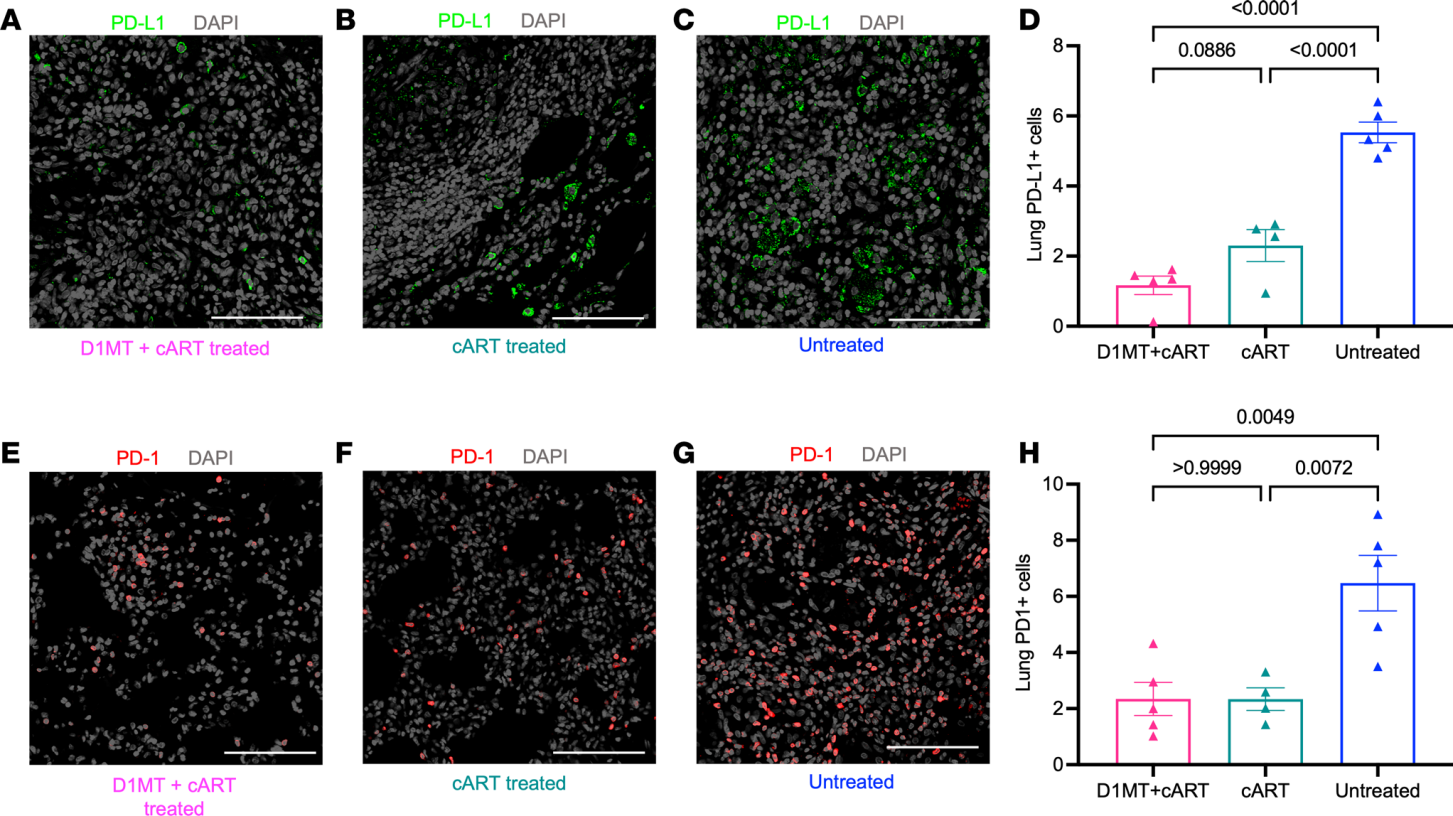
PD-L1 and PD-1 expression. (**A**–**C** and **E**–**G**) Lung sections were stained for PD-L1 (green), PD-1 (red), and DAPI (gray). The representative confocal images of the lung of the D1MT+cART-treated (**A** and **E**), cART-treated (**B** and **F**), and untreated (**C** and **G**) macaques, captured at ×20 magnification. (**D** and **H**) Graph depicting percentages of PD-L1^+^ and PD-1^+^ cells in lungs of D1MT+cART-treated, cART-treated, and untreated macaques. Scale bars: 100 μm. *P* values are indicated above the plots as obtained from 1-way ANOVA (**D** and **H**). Data are represented as mean ± SEM.

Since IDO and PD-L1 expression has been shown to be highly correlated in a variety of myeloid cell populations in human TB granulomas (8), we next studied the expression of activation marker PD-1 and its ligand inhibitor PD-L1 in the lungs of the different groups of RMs (Figure 11). The frequency of PD-L1^+^ cells in the lungs was significantly reduced in both cART-treated groups relative to untreated groups (~6%), but it was further reduced in the D1MT+cART (~1%) relative to the cART group (~2.25%); this difference approached the threshold of significance (*P* = 0.0886) (Figure 11, A–D). A similar decline in the frequency of PD-1^+^ cells in the lungs of the 3 groups was also observed (Figure 11, E–H), although only the difference between the 2 treatment groups (~2.2% and ~2.1%) relative to untreated group (~6.5%) was significant.

Soluble CD14 (sCD14) and sCD163 are known markers of chronic immune activation that are negatively correlated with CD4^+^ T cell reconstitution and treatment response (39, 40). We therefore studied the effect of cART, and additionally of D1MT, on sCD14 and sCD163 levels in both the peripheral blood as well as the luminal compartment in *M. tuberculosis*/SIV–coinfected RMs (Supplemental Figure 9). In the BAL, the levels of sCD14 remained at baseline in all groups after SIV coinfection but gradually increased such that significantly higher levels of sCD14 were present in each of the 3 groups at endpoint (Supplemental Figure 9A). While cART alone did not significantly reduce the concentrations of sCD14 in BAL, the inclusion of D1MT significantly reduced sCD14 levels relative to the cART-only group (*P* = 0.023) (Supplemental Figure 9A). A similar trend was observed in plasma where sCD14 levels in untreated *M. tuberculosis*/SIV–coinfected RMs were significantly higher than the 2 treatment groups (Supplemental Figure 9C). sCD163 levels were then only measured at the endpoint. While these concentrations increased in both the BAL (Supplemental Figure 9C) and plasma (Supplemental Figure 9D) for untreated RMs, relative to the 2 treatment groups, the differences were only statistically significant in plasma (Supplemental Figure 9D). Together, the results clearly show a significant reduction in chronic immune activation measures in D1MT+cART-treated relative to cART-only–treated RMs.

## Discussion

Most immunocompetent individuals control *M. tuberculosis*–infection, but coinfection with HIV increases the risk of progressing to TB by > 20-fold (41). While cART decreases TB reactivation rates (42), incidences remains 4- to 7-fold higher in PLHIV on cART relative to HIV-uninfected individuals (43–45). Thus, ART does not fully restore the immune control of *M. tuberculosis* infection in PLHIV. Despite the effective control of viral replication by ART, HIV/SIV-induced chronic immune activation persists, due to bystander T/B cell activation by inflammatory cytokines, microbial translocation, and turnover of inflammatory macrophages that express immunosuppressive molecules like IDO (40). Chronic immune activation in the lungs of *M. tuberculosis*/SIV–coinfected RMs correlates with the presence of SIV (38). Most *M. tuberculosis*/SIV–coinfected RMs reactivate LTBI, with lung pathology (38), signatures of chronic immune activation (46), and recruitment/high turnover of IMs in the lung (38). RMs with TB but not asymptomatic *M. tuberculosis* infection are characterized by increased recruitment of pDCs — the primary source of type I IFN (4). Immune activation upregulates IDO expression downstream of type I IFN (47) in IMs (4). A population of IDO^+^, IFI/IFIT^+^, and CXCL9-11^+^ IMs in the lung correlates strongly with TB (4) and reactivation due to coinfection (38, 48). We now show that inclusion of D1MT adjunctive to cART significantly reduces the recruitment of pDCs to the lungs (Figure 10, A–D), lowering type I IFN signaling in pDCs (Figure 10, I–L) as well as IMs (Figure 10, M–P). Hence, IDO links chronic immune activation and TB reactivation (49).

Since HDTs against TB are most likely to be deployed in the settings of multidrug-resistant TB (MDR-TB) and HIV coinfection, here, we focused on the effect of IDO inhibition in the *M. tuberculosis*/HIV coinfection setting in our RM model, adjunctive to cART. Interest in HDTs (50) is driven by the need to shorten conventional TB therapy to reduce MDR-TB and by rapid progression of TB disease in *M. tuberculosis*/HIV coinfection (51). HDTs are most likely to be deployed for use in MDR-patients with TB and those who are coinfected. Since IDO is linked to chronic immune activation and TB reactivation, it is an important HDT target for TB/HIV. In the current study, inclusion of D1MT in *M. tuberculosis*/SIV–coinfected, cART-treated RMs did not result in an increase in clinical markers of TB or HIV disease, or lung pathology. This is important for 2 reasons. D1MT promotes Th1 responses by increasing T cell proliferation and activation. D1MT treatment could, therefore, lead to enhanced tissue pathology. Our results show, however, that in vivo IDO inhibition can be achieved by D1MT treatment concurrently with cART without increased lung pathology or clinical TB in RMs. Instead, we report that inhibition of IDO decreases chronic immune activation. D1MT+cART-treated RMs were characterized by the presence of an optimal immune response to *M. tuberculosis*, despite SIV coinfection: one characterized by an increased recruitment of CD4^+^ and CD8^+^ T cells to the lung, and their enhanced activation and proliferation phenotype, in the absence of chronic immune activation characterized by IDO^+^ myeloid cells. Hence, D1MT treatment is safe and efficacious in RMs and can be effectively used along with cART. While most studies on the effect of IDO inhibition on lymphocytes have focused on CD4^+^ T cells and demonstrate their increased activation/proliferation phenotype upon D1MT treatment, this effect has also been described for CD8^+^ T cells in both animals (27) and humans (28). This is not surprising since Trp is not only an essential amino acid but also the rarest and is required for the rapid proliferation of both CD4^+^ and CD8^+^ T cells. Suppressing IDO activity promotes the generation of both memory and effector memory CD8^+^ T cells and increases their proliferative capacity, while the overexpression of IDO attenuated their generation and proliferation (52). Our current results suggest a role for CD8^+^ T cells in potentially mediating control of TB reactivation. Further studies are required to test this hypothesis.

Chronic immune activation persists in SIV-infected and *M. tuberculosis*/SIV–coinfected RMs, even after cART. Our results clearly show that the inclusion of D1MT not only inhibits IDO levels but significantly reduces the recruitment of IMs to the lung, which is a key step in SIV-mediated reactivation of *M. tuberculosis* infection (48). It is very likely that this reduction in immune activation is responsible for enhanced *M. tuberculosis*–specific Th1 and activation responses in CD4^+^ and CD8^+^ T cells recruited to the lung, even in the absence of *M. tuberculosis* antigens. It is important to note, however, that activated, proliferative CD4^+^ T cells could also serve as more targets for SIV(HIV) replication. In our study, cART was studied for 11–12 weeks, and we did not observe increased SIV viremia in the peripheral blood or BALF or in the lung tissue. Long-term effects of D1MT treatment on proliferating CD4^+^ T cells and viral loads must, however, be studied in RMs before IDO inhibition can be attempted in a human TB setting. Additionally, while we have demonstrated the adjunctive effect of IDO inhibition with cART here, and with anti-TB therapy (ATT) earlier (19), we would, in the future, need to show the effectiveness of this HDT strategy adjunctive to simultaneously administered ATT/cART, since clinical recommendations require concurrent treatment of most PLHIV with ATT/cART. Our current and previous results do, however, place D1MT as a leading HDT candidate for TB, especially since our results have been obtained in the human-like NHP model of TB, and *M. tuberculosis*/HIV coinfection. While at least 2 groups have developed NHP models of *M. tuberculosis/*SIV coinfection (21, 53), concurrent treatment of ATT/cART in these models has not yet been reported.

## Methods

### Sex as a biological variable.

Sex was not considered as a biological variable.

### Animal infections and treatments.

Twenty Indian-origin RMs used in this study were infected with a low dose (~10 CFUs) of *M. tuberculosis* CDC1551 via aerosol as described previously(23). All RMs were monitored weekly for serum CRP levels, changes in body weight and temperatures, and changes in cellular biochemistry (CBC) and hematology. Upon the establishment of LTBI, the RMs were infected with 300 TCID_50_ SIVmac_239_ via i.v. route at week 9 after *M. tuberculosis* infection. SIV infection was confirmed with quantitative PCR (qPCR). *M. tuberculosis*/SIV–coinfected RMs were divided into 3 groups: 5 in the D1MT+cART group (current study), 4 in the cART group that were historical controls (2), and 11 untreated, of which 3 were from the current study and 8 were historical controls (3). At 2 weeks after SIV infection, the D1MT+cART group was treated with D1MT daily for 4 weeks (week 11 to week 15), and cART treatment of both D1MT+cART and cART groups continued from week 11 until the endpoint, following which necropsies were scheduled. Study demographics are provided in Supplemental Table 1.

### cART/D1MT regimen.

cART was administered at week 11 (week 2 after SIV) as described earlier (2). D1MT (45 mg/kg of body weight) was administered daily via oral route for 4 weeks (week 11 to week 15) as described previously (19).

### Viral load and bacterial burden measurement.

SIV measurements in plasma and BAL supernatant were performed as described earlier (2, 3, 20, 22, 23). For SIV measurement, the lung was homogenized in Trizol, followed by RNA extraction and qPCR for determination of SIV copy numbers. *M. tuberculosis* CFU measurements in BAL, lung, granulomas, and other tissues were performed as described earlier (2, 3, 20, 22, 23).

### PET/CT.

18-FDG PET/CT was performed as described earlier (19, 23), at the following time points: week 6 (pre-SIV), week 10/11 (pre-treatment), week 16 (D1MT treatment end), and week 22/endpoint (cART treatment end). Briefly, the RMs were longitudinally scanned using both the PET and the CT modalities of a Mediso’s LFER150 PET-CT scanner operational within our Animal Biosafety Level-3 (ABSL3) facility at different time points, using a breath-hold technique, starting from before *M. tuberculosis* infection with the last scan just prior to necropsy (23). Animals were anesthetized and intubated under supervision of our veterinarians as per approved IACUC protocols, as previously described. All the animals received an i.v. injection of 5 mCi dose of 18F-FDG, procured from Cardinal Health. The single field of view (FOV) and/or double FOV lung CT scans were performed (23). Images were visualized using Interview Fusion 3.03 (Mediso) and reconstructed using Nucline NanoScan LFER 1.07 (Mediso) as previously described (19, 23). The lung segmentation, volumetric and SUV analysis was performed using Vivoquant 4.0 (Invicro) (24). The maximum standardized uptake value (SUVmax) of the 18F-FDG in the lungs of the TB-infected RMs, usually seen in the lung lesions, was represented as lung SUVmax (19, 23). Granuloma count was performed by identifying and counting heterogenous TB lesions manually (19, 23).

### High-parameter flow cytometry.

Immune dynamics were measured using high-parameter flow cytometry T cell panels as described earlier (3, 19, 23, 54, 55). Antigen-specific flow cytometry–based ICCS was performed as described earlier (54). Antibodies and reagents are provided in Supplemental Table 2. Gating strategies for flow cytometry and antigen-specific assays are described in Supplemental Figure 2.

### Lung pathology evaluation.

Pathological measurements were performed by board-certified veterinary pathologists using methods described in detail earlier (19, 23). Briefly, lung tissue was collected stereologically at necropsy, fixed and stained with H&E using standard methods for histologic analysis, and scanned with Zeiss Axioscan Z1 at ×40 magnification, in a blinded manner. TB pathology was calculated as an average for each animal in the study based on scores assigned to multiple lung sections (22). In case of lungs, 18–24 random gross samples were selected from serial sections of lung using an overlay grid with 18.5 mm point spacing. The same grid was utilized on scanned microscopic images at ×2.5 magnification to quantify total lung, hemorrhage, edema, cellular infiltration, lymphoid aggregates, and necrosis (35). Twenty adjacent sites were pooled for analysis by culture (35). The digital slides were analyzed using an optimized tissue classifier in HALO software v3.6 (Indica Labs) to quantify the percentage of lung affected.

### Immunofluorescence and IHC.

IHC staining was performed as described earlier (18) on the FFPE lung sections from the D1MT+cART, cART-only, and untreated groups. The lung sections were stained for IDO, macrophages, pDCs, CD4, CD8, IFN-α, PD-1, PD-L1 markers. The whole slides were scanned using Zeiss Axiscan Z1 and quantified using HALO software. Immunofluorescence staining for Kyn was performed on BAL cell cytospins as described previously (19) from pre-D1MT treatment (week 11) and post-D1MT treatment (week 15) time points, to examine IDO activity. The representative images were captured using Zeiss LSM-800 confocal microscope and analyzed using ImageJ (Fiji; NIH) to quantify Kyn^+^ cells in BAL. Antibodies used in immunofluorescence and IHC experiments are listed in Supplemental Table 3.

### Statistics.

Statistical analysis was performed using 1- and 2-way ANOVA with Šidák’s or Tukey’s multiple corrections in GraphPad Prism (version 9.4.1). Contingency χ^2^ and Fisher’s exact test were used to measure extent of sterility among the experimental groups. A *P* value of less than 0.05 was considered statistically significant. Data are presented as mean ± SEM.

### Study approval.

All procedures performed on NHPs were either approved by the Texas Biomed Biosafety Committee and its IACUC or by Tulane National Primate Research Center’s IACUC and Tulane University’s Institutional Biosafety Committee.

### Data availability.

Values for all data points in graphs are reported in the Supporting Data Values file.

## Author contributions

SM, DK and BS optimized experimental design; BS, RS, RE, GR, GA, VS, EJD, XA and DKS performed research; VS, EJD, SHU and XA provided expertise in specialized pathology, veterinary, and imaging techniques; BS, RS, and SM performed data analysis; SM wrote the paper with inputs from BS and DK; and SM, DKS, and DK provided funding.

## Supplementary Material

Supplemental data

Supporting data values

## Version 1 08/08/2024

Electronic publication

## References

[1] Sharan R (2020). Chronic immune activation in TB/HIV co-infection. Trends Microbiol.

[2] Sharan R (2022). Antiretroviral therapy timing impacts latent tuberculosis infection reactivation in a tuberculosis/simian immunodeficiency virus coinfection model. J Clin Invest.

[3] Ganatra SR (2020). Anti-retroviral therapy does not reduce tuberculosis reactivation in a tuberculosis-HIV co-infection model. J Clin Invest.

[4] Esaulova E (2021). The immune landscape in tuberculosis reveals populations linked to disease and latency. Cell Host Microbe.

[5] Singh DK (2022). Myeloid cell interferon responses correlate with clearance of SARS-CoV-2. Nat Commun.

[6] Akter S (2022). Mycobacterium tuberculosis infection drives a type I IFN signature in lung lymphocytes. Cell Rep.

[8] McCaffrey EF (2022). The immunoregulatory landscape of human tuberculosis granulomas. Nat Immunol.

[9] Collins JM (2020). Tryptophan catabolism reflects disease activity in human tuberculosis. JCI Insight.

[10] Isa F (2018). Mass spectrometric identification of urinary biomarkers of pulmonary tuberculosis. EBioMedicine.

[11] Tornheim JA (2021). The kynurenine/tryptophan ratio is a sensitive biomarker for the diagnosis of pediatric tuberculosis among Indian children. Front Immunol.

[12] Taylor MW, Feng GS (1991). Relationship between interferon-gamma, indoleamine 2,3-dioxygenase, and tryptophan catabolism. FASEB J.

[13] Mehra S (2010). Transcriptional reprogramming in nonhuman primate (rhesus macaque) tuberculosis granulomas. PLoS One.

[14] Mehra S (2013). Granuloma correlates of protection against tuberculosis and mechanisms of immune modulation by Mycobacterium tuberculosis. J Infect Dis.

[15] Kondrikov D (2020). Kynurenine inhibits autophagy and promotes senescence in aged bone marrow mesenchymal stem cells through the aryl hydrocarbon receptor pathway. Exp Gerontol.

[16] Fallarino F (2003). T cell apoptosis by kynurenines. Adv Exp Med Biol.

[17] Gautam US (2018). In vivo inhibition of tryptophan catabolism reorganizes the tuberculoma and augments immune-mediated control of *Mycobacterium tuberculosis*. Proc Natl Acad Sci U S A.

[18] Singh B (2021). Myeloid-derived suppressor cells mediate T cell dysfunction in nonhuman primate TB granulomas. mBio.

[19] Singh B (2023). Inhibition of indoleamine dioxygenase leads to better control of tuberculosis adjunctive to chemotherapy. JCI Insight.

[20] Bucsan AN (2019). Mechanisms of reactivation of latent tuberculosis infection due to SIV coinfection. J Clin Invest.

[21] Mehra S (2011). Reactivation of latent tuberculosis in rhesus macaques by coinfection with simian immunodeficiency virus. J Med Primatol.

[22] Foreman TW (2016). CD4^+^ T-cell-independent mechanisms suppress reactivation of latent tuberculosis in a macaque model of HIV coinfection. Proc Natl Acad Sci U S A.

[23] Sharan R (2022). Isoniazid and rifapentine treatment effectively reduces persistent M. tuberculosis infection in macaque lungs. J Clin Invest.

[24] Gough M (2022). Peripheral blood markers correlate with the progression of active tuberculosis relative to latent control of *Mycobacterium tuberculosis* infection in macaques. Pathogens.

[25] Boasso A (2007). Regulatory T-cell markers, indoleamine 2,3-dioxygenase, and virus levels in spleen and gut during progressive simian immunodeficiency virus infection. J Virol.

[26] Shanmugasundaram U (2020). Pulmonary Mycobacterium tuberculosis control associates with CXCR3- and CCR6-expressing antigen-specific Th1 and Th17 cell recruitment. JCI Insight.

[27] Liu H (2009). Reduced cytotoxic function of effector CD8^+^ T cells is responsible for indoleamine 2,3-dioxygenase-dependent immune suppression. J Immunol.

[28] Sorensen RB (2009). The immune system strikes back: cellular immune responses against indoleamine 2,3-dioxygenase. PLoS One.

[29] Merlo LMF (2022). Impact of IDO1 and IDO2 on the B cell immune response. Front Immunol.

[30] Das A (2011). Simian immunodeficiency virus infection in rhesus macaques induces selective tissue specific B cell defects in double positive CD21+CD27+ memory B cells. Clin Immunol.

[31] Foreman TW (2022). CD4 T cells are rapidly depleted from tuberculosis granulomas following acute SIV co-infection. Cell Rep.

[32] Cartwright EK (2016). Initiation of antiretroviral therapy restores CD4^+^ T memory stem cell homeostasis in simian immunodeficiency virus-infected macaques. J Virol.

[33] Mellor AL, Munn DH (2004). IDO expression by dendritic cells: tolerance and tryptophan catabolism. Nat Rev Immunol.

[34] de Araujo EF (2017). The IDO-AhR axis controls Th17/Treg immunity in a pulmonary model of fungal infection. Front Immunol.

[35] Kaushal D (2015). Mucosal vaccination with attenuated Mycobacterium tuberculosis induces strong central memory responses and protects against tuberculosis. Nat Commun.

[36] Slight SR (2013). CXCR5^+^ T helper cells mediate protective immunity against tuberculosis. J Clin Invest.

[37] Swanson RV (2023). Antigen-specific B cells direct T follicular-like helper cells into lymphoid follicles to mediate Mycobacterium tuberculosis control. Nat Immunol.

[38] Kuroda MJ (2018). High turnover of tissue macrophages contributes to tuberculosis reactivation in simian immunodeficiency virus-infected rhesus macaques. J Infect Dis.

[39] Paiardini M, Muller-Trutwin M (2013). HIV-associated chronic immune activation. Immunol Rev.

[40] Sharan R (2020). Chronic immune activation in TB/HIV co-infection. Trends Microbiol.

[41] Sonnenberg P (2005). How soon after infection with HIV does the risk of tuberculosis start to increase? A retrospective cohort study in South African gold miners. J Infect Dis.

[42] Velasco M (2009). Effect of simultaneous use of highly active antiretroviral therapy on survival of HIV patients with tuberculosis. J Acquir Immune Defic Syndr.

[43] Walker NF (2013). HIV-1 and the immune response to TB. Future Virol.

[44] Sutherland JS (2010). Polyfunctional CD4(+) and CD8(+) T cell responses to tuberculosis antigens in HIV-1-infected patients before and after anti-retroviral treatment. J Immunol.

[45] Gupta A (2012). Tuberculosis incidence rates during 8 years of follow-up of an antiretroviral treatment cohort in South Africa: comparison with rates in the community. PLoS One.

[46] Bucsan AN (2019). Mechanisms of reactivation of latent tuberculosis infection due to SIV co-infection. J Clin Invest.

[47] Puccetti P (2007). On watching the watchers: IDO and type I/II IFN. Eur J Immunol.

[48] Khan N, Divangahi M (2018). Mycobacterium tuberculosis and HIV Coinfection Brings Fire and Fury to Macrophages. J Infect Dis.

[49] Kaushal D (2023). Immune responses in lung granulomas during Mtb/HIV co-infection: implications for pathogenesis and therapy. Pathogens.

[50] Tobin DM (2015). Host-directed therapies for tuberculosis. Cold Spring Harb Perspect Med.

[51] Maiga M (2012). Successful shortening of tuberculosis treatment using adjuvant host-directed therapy with FDA-approved phosphodiesterase inhibitors in the mouse model. PLoS One.

[52] Liu Z (2007). Suppression of memory CD8 T cell generation and function by tryptophan catabolism. J Immunol.

[53] Mattila JT (2011). Simian immunodeficiency virus-induced changes in T cell cytokine responses in cynomolgus macaques with latent Mycobacterium tuberculosis infection are associated with timing of reactivation. J Immunol.

[54] Sharan R (2021). Characterizing early T cell responses in nonhuman primate model of tuberculosis. Front Immunol.

[55] Singh DK (2021). Responses to acute infection with SARS-CoV-2 in the lungs of rhesus macaques, baboons and marmosets. Nat Microbiol.

