# Supplementary material for: Indoleamine-2,3-dioxygenase inhibition improves immunity and is safe for concurrent use with cART during Mtb/SIV coinfection
Source: JCI Insight. 2024 Jul 2;9(15):e179317. doi: 10.1172/jci.insight.179317 (PMC11383603; doi:10.1172/jci.insight.179317)
Supplement: Supplemental data [file jciinsight-9-179317-s245.pdf]

Figure S1

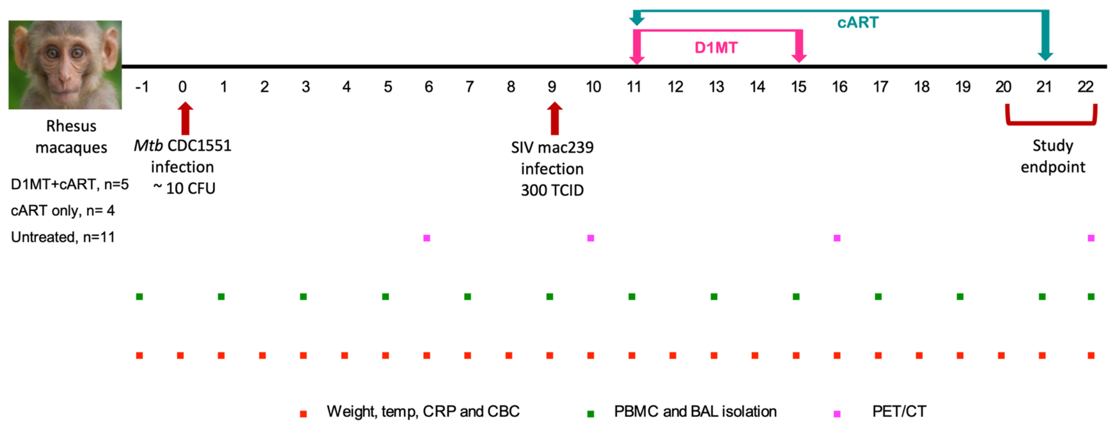

Supplementary Figure 1. The study timeline showing the details about the infection, treatment groups, period of treatment and the procedures performed.

Figure S2

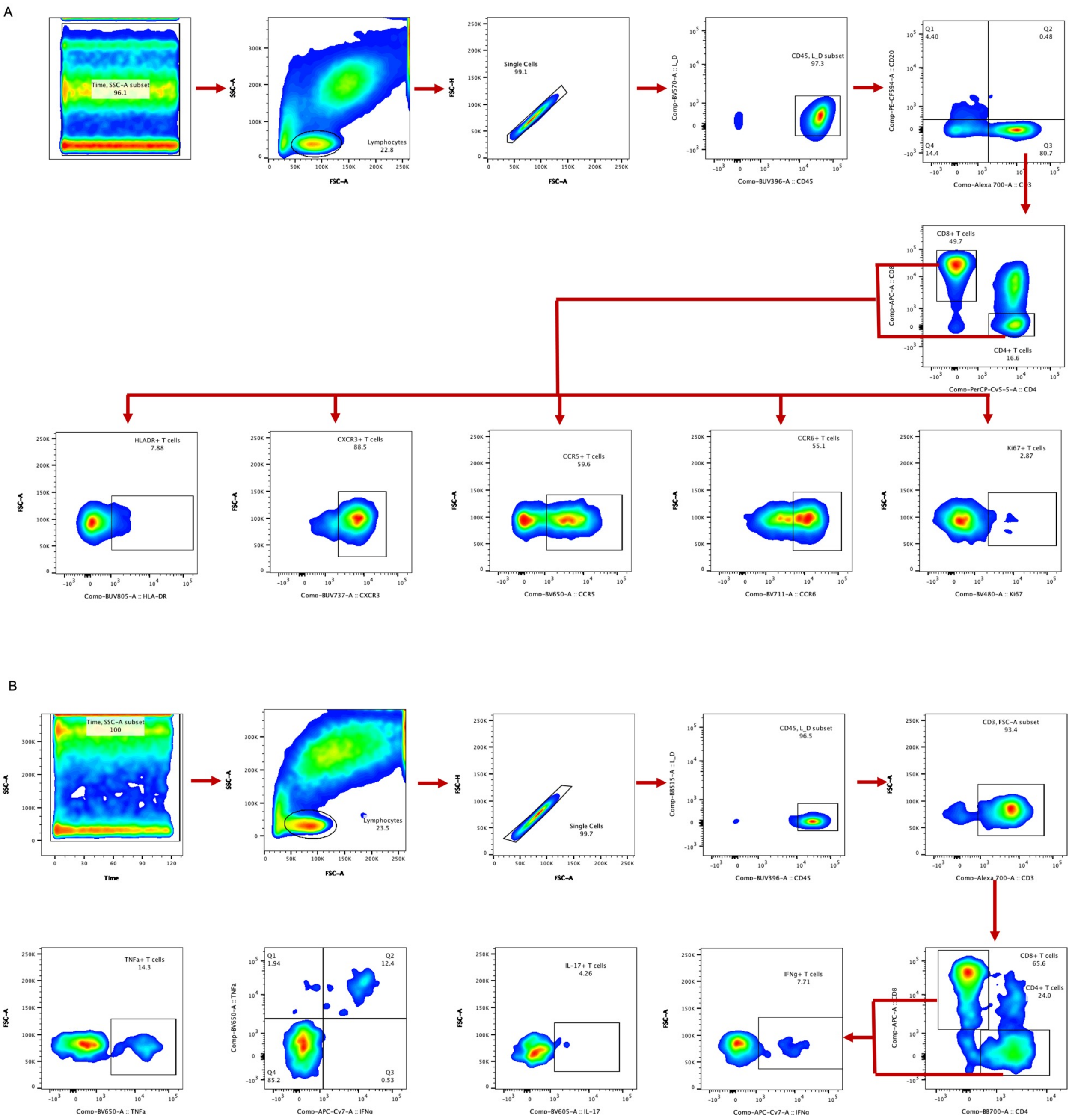

Supplementary Figure 2. Representative gating strategy for **(A)** T-cell phenotyping and **(B)** Mtb specific responses.

Figure S3

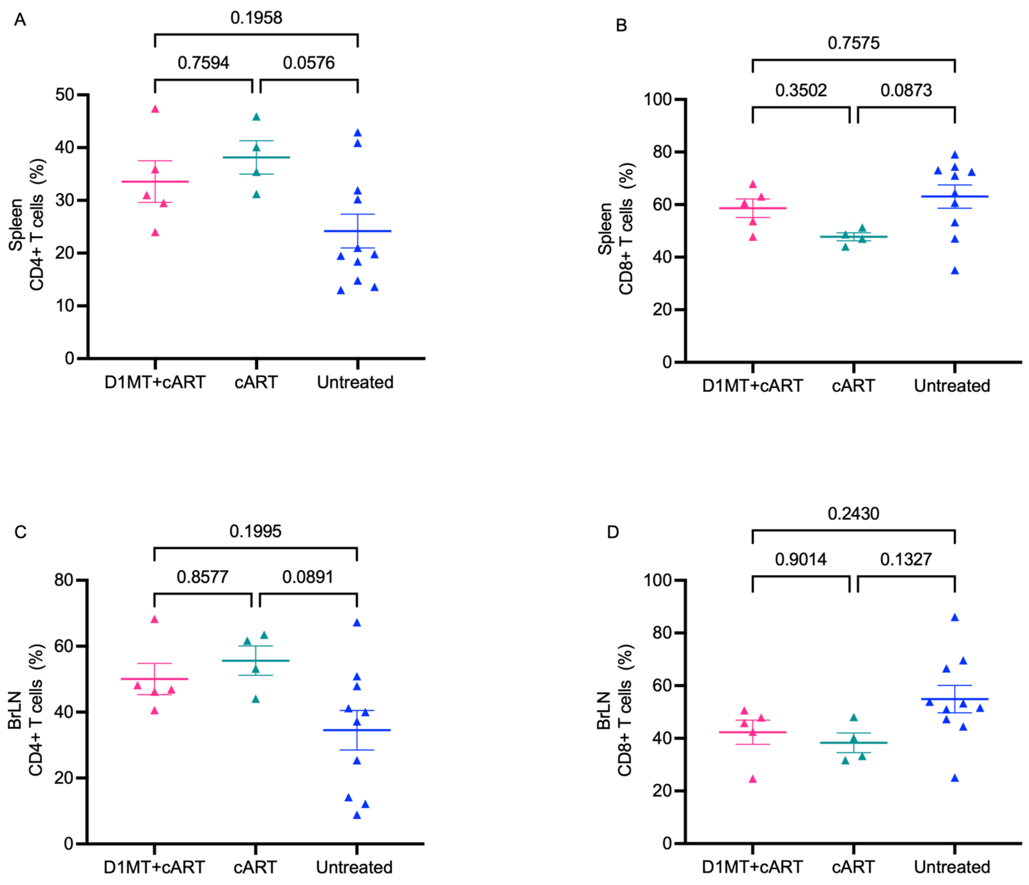

Supplementary Figure 3. Graphs depicting percentages of CD4^{+} T **(A)** and CD8^{+} T **(B)** cells in spleen. Graph showing percentages of CD4^{+} T **(C)** and CD8^{+} T **(D)** in bronchial lymph nodes. *P* values are indicated above the plot as obtained from one-way ANOVA. Data are represented as mean ± SEM.

Figure S4

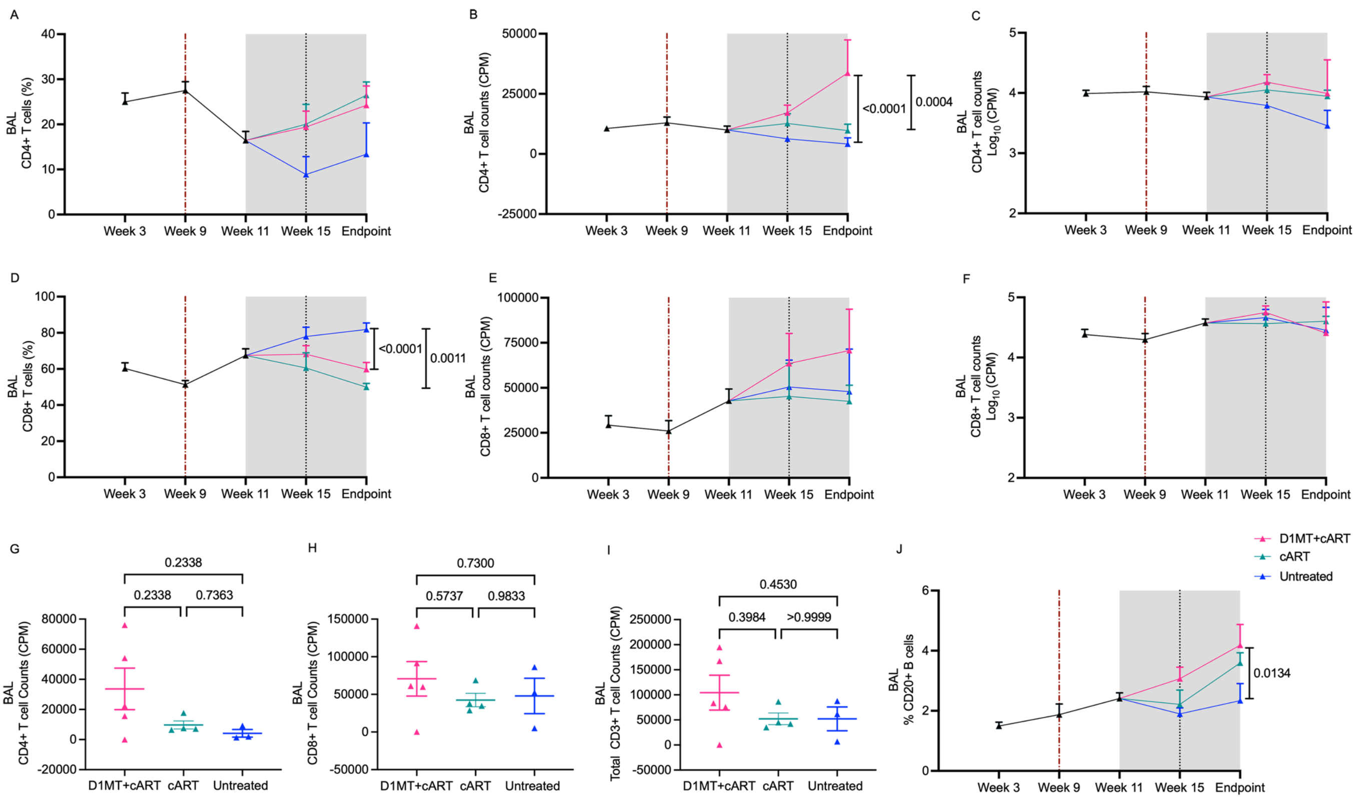

Supplementary Figure 4. Graphical representation of data obtained from flow-cytometry experiments depicting the percentages of total $CD4^+$ T cells **(A)**, $CD8^+$ T cells **(D)** and CD20+ B cells **(J)** in BAL collected at various time-points of the study. Graph showing changes in $CD4^+$ T cell counts per million (CPM) **(B),** log $CD4^+$ T CPM **(C),** $CD8^+$ T cell counts per million **(E),** log $CD8^+$ T CPM **(F)** in BAL at different points of the study. Graph showing $CD4^+$ T CPM **(G)**, $CD8^+$ T **(H)** and total $CD3^+$ T cells **(I)** in BAL at endpoint. *P* values are indicated above the plot as obtained from two-way ANOVA **(A-F, J)** and one-way ANOVA **(G-I)**. Data are represented as mean ± SEM.

Figure S5

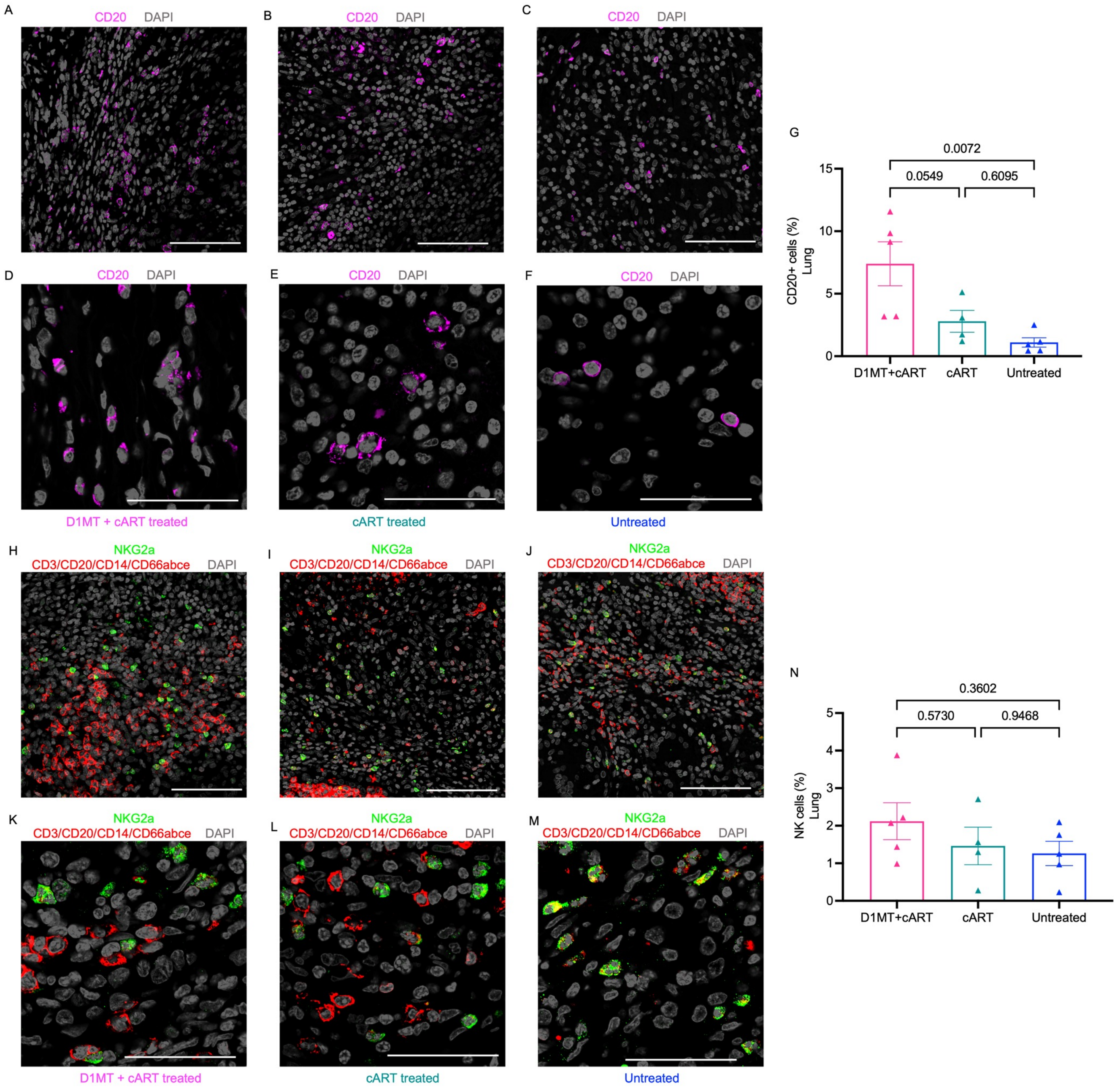

Supplementary Figure 5. B-cell and NK cell dynamics. The lung sections were stained for B-cells (CD20+) and NK cells (NKG2a+ CD3-, CD20-, CD14-, CD66abce-). Shown are the representative confocal images of the lung of the D1MT+cART treated **(A, D, H, K)**, cART treated **(B, E, I, L)** and untreated **(C, F, J, M)** macaques, captured at 20X magnification **(A-C, H-J)** and 63X magnification **(D-F, K-M)**. Graph depicting the percentages of **(D)** CD20+ B cells, and **(N)** NK cells in lungs of D1MT+cART treated, cART treated and untreated macaques. Scale bars, 100 μm (20X magnification; **A**, **B**, **C**, **H**, **I** and **K**); 50 μm (63X magnification; **D**, **E**, **F**, **K**, **L** and **M**). *P* values are indicated above the plots as obtained from one-way ANOVA **(G, N).** Data are represented as mean ± SEM.

Figure S6

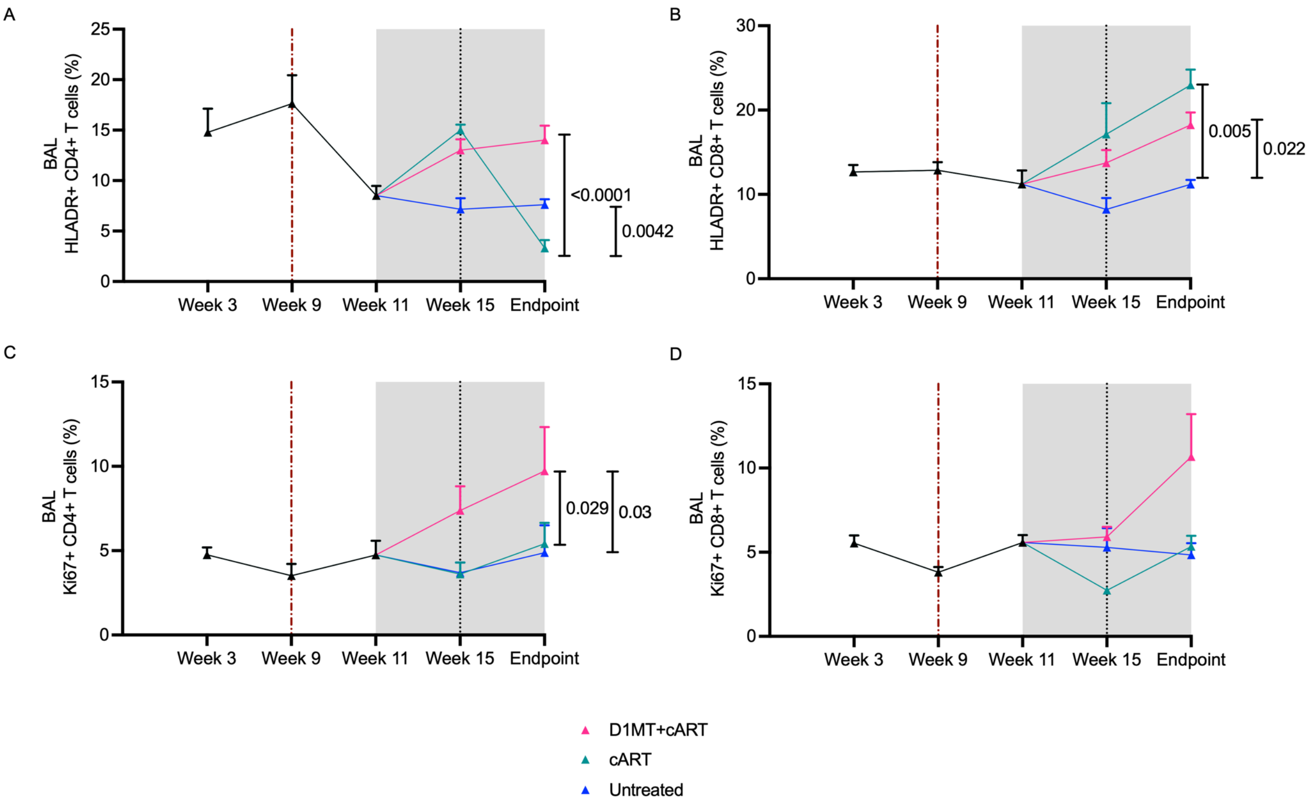

Supplementary Figure 6. Graphical representation of data obtained from flow-cytometry experiments depicting the percentages of HLADR^{+} CD4^{+} T cells **(A)**, HLADR^{+} CD8^{+} T cells **(B),** Ki67^{+} CD4^{+} T cells **(C)** and Ki67^{+} CD8^{+} T cells **(D)** in BAL collected at various time-points of the study. *P* values are indicated above the plot as obtained from two-way ANOVA. Data are represented as mean ± SEM.

Figure S7

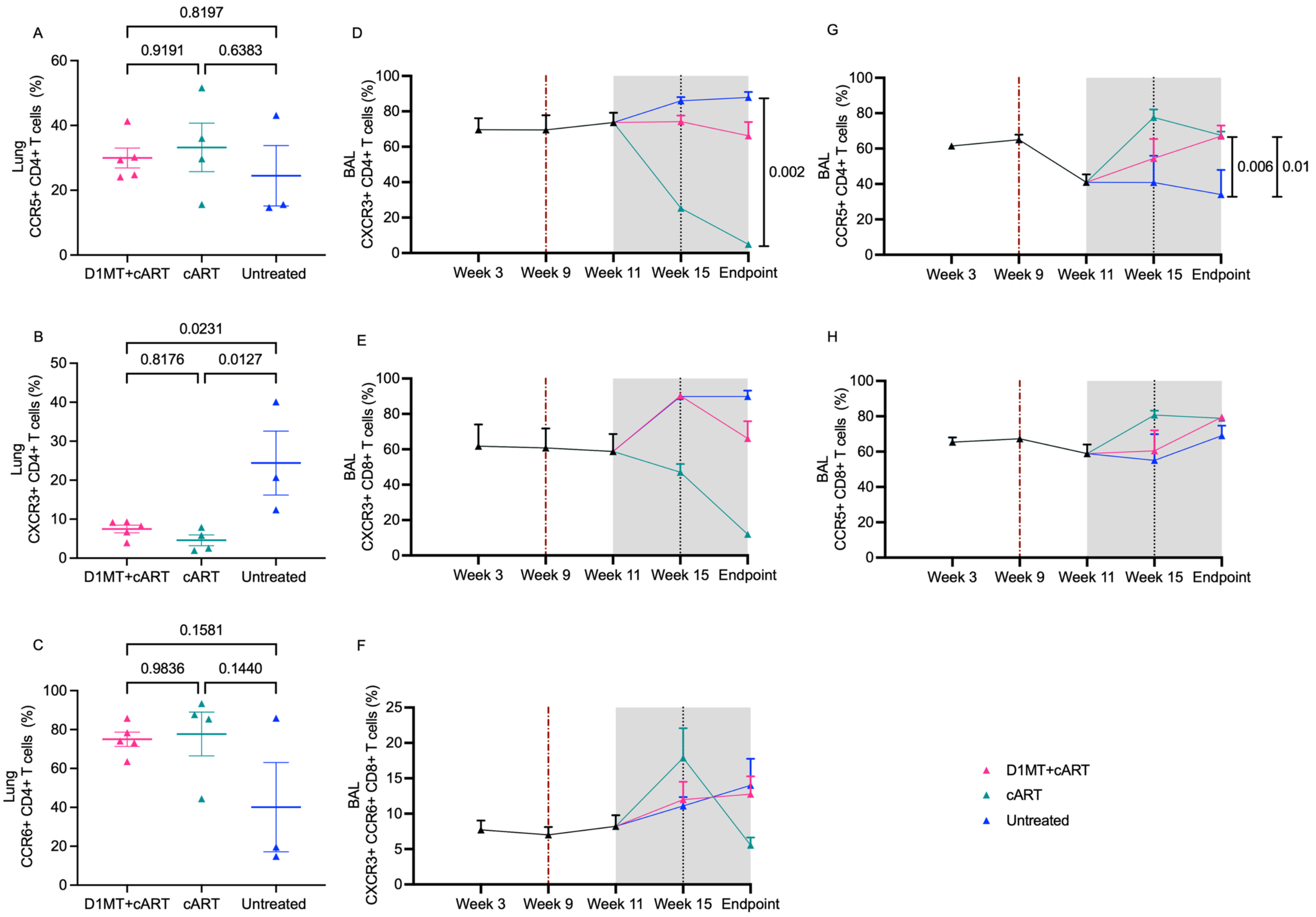

Supplementary Figure 7. Graphs showing percentages of $CCR5^+$, $CXCR3^+$ and $CCR6^+$ $CD4^+$ T cells in lungs **(A, B, C)**, respectively. Percentages of $CXCR3^+CD4^+$ T cells **(D),** $CXCR3^+CD8^+$ T cells **(E)** $CXCR3^+CCR6^+CD8^+$ T cells **(F)**, $CCR5^+CD4^+$ T cells **(G)** and $CCR5^+CD8^+$ T cells **(H)** in BAL at various timepoints in D1MT+cART treated, cART treated and untreated groups. *P* values are indicated above the plot as obtained from one-way ANOVA **(A, B, C)** and two-way ANOVA **(D, E, F, G, H).** Data are represented as mean ± SEM.

Figure S8

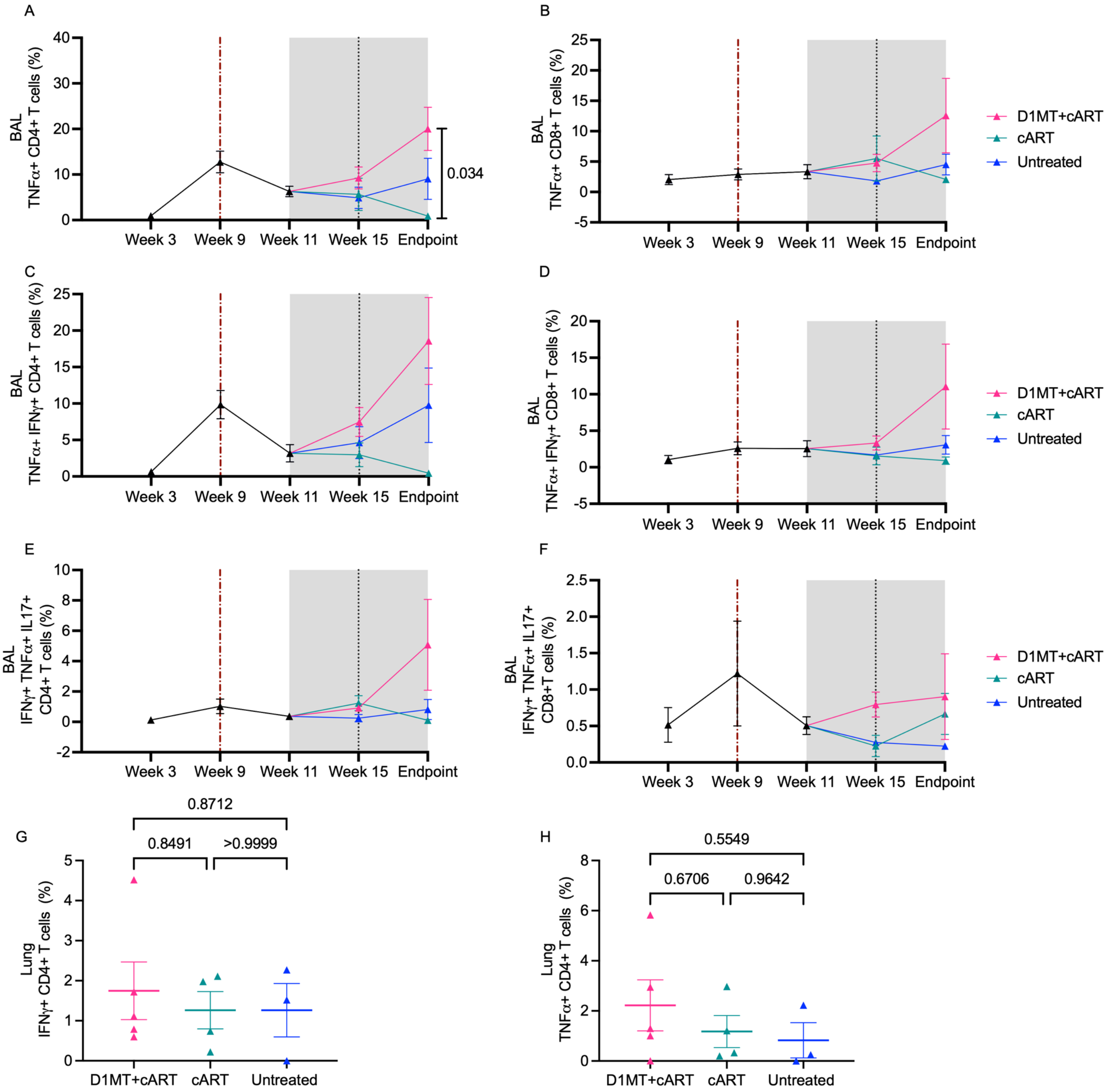

Supplementary Figure 8. Graphs showing percentages of Mtb CW specific TNFα^+ CD4$^+$ T cells **(A)** and TNFα^+ CD8$^+$ T cells **(B),** IFNγ^+ TNFα^+ CD4$^+$ T cells **(C)** and IFNγ^+ TNFα^+ CD8$^+$ T cells **(D),** IFNγ^+ TNFα^+ IL 17$^+$ CD4$^+$ T cells **(E)** and IFNγ^+ TNFα^+ IL 17$^+$ CD8$^+$ T cells **(F)** in BAL of D1MT+cART and untreated RMs at different timepoints of the study. Mtb CW specific IFN$\boldsymbol{\gamma}^+$ CD4$^+$ T cell **(G)** and TNFα^+ CD4$^+$ T cell **(H)** percentages in lungs of untreated, D1MT+cART and cART groups. *P* values are indicated above the plot as obtained from two-way ANOVA **(A, B, C, D, E, F)** and one-way ANOVA **(G, H).** Data are represented as mean ± SEM.

Figure S9

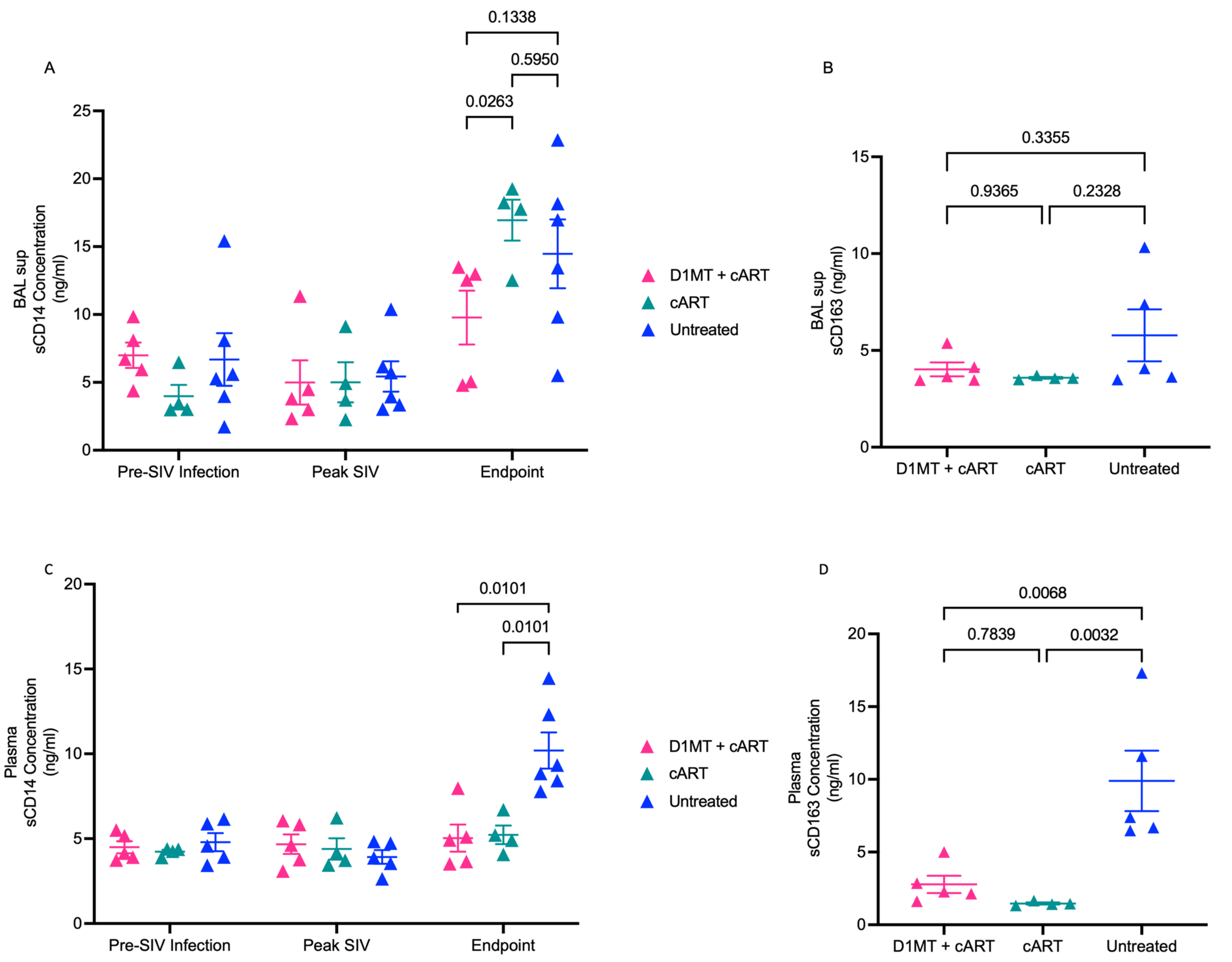

Supplementary Figure 9. Soluble CD14 and CD163. The concentrations of sCD14 and sCD163 were determined by ELISA in BAL supernatant and plasma of three groups of macaques. Graphs showing concentration of sCD14 in BAL sup **(A)** and plasma **(C)** at different timepoints. Graphs depicting sCD163 present in BAL sup **(B)** and plasma **(D)** at endpoint in untreated, D1MT+cART and cART groups. *P* values are indicated above the plot as obtained from two-way ANOVA **(A, C)** and one-way ANOVA **(B, D).** Data are represented as mean ± SEM.
